# Supplementary figures and images for: Adenomatous Polyposis Coli loss controls cell cycle regulators and response to paclitaxel in MDA-MB-157 metaplastic breast cancer cells
Source: PLoS One. 2021 Aug 9;16(8):e0255738. doi: 10.1371/journal.pone.0255738 (PMC8351968; doi:10.1371/journal.pone.0255738)

A

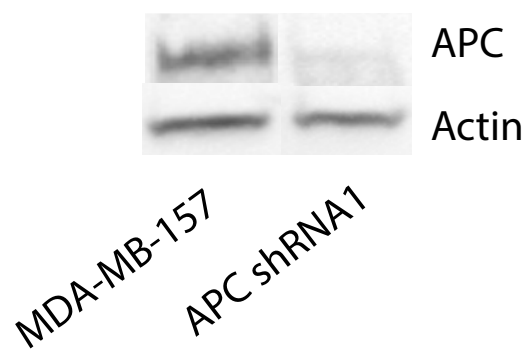

B

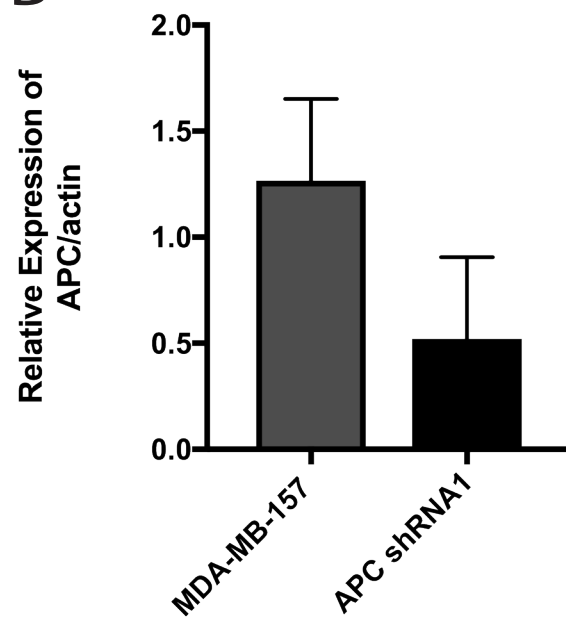

Supplement: S1 Fig — (A) Untreated cell lysates were probed for APC expression and representative western blot is shown. (B) Bar graph displays average (n = 3) expression of APC relative to actin in MDA-MB-157 and APC shRNA1 cells. (PDF) [file pone.0255738.s001.pdf]

A

Propidium Iodide (PI)

MDA-MB-157 control

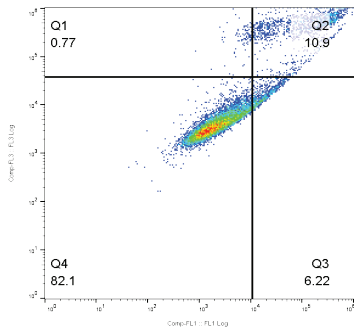

APC shRNA1 control

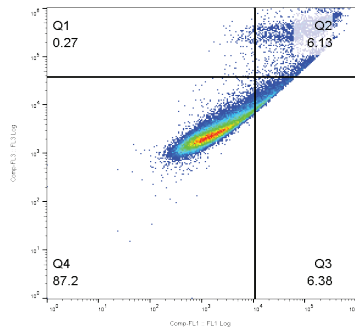

MDA-MB-157 PTX

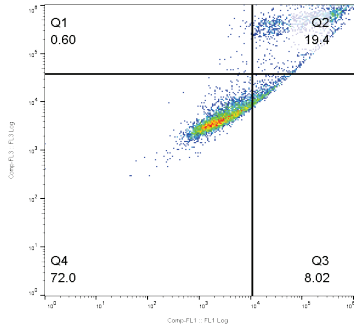

APC shRNA1 PTX

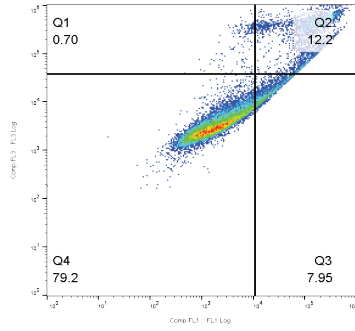

Annexin V-488

B

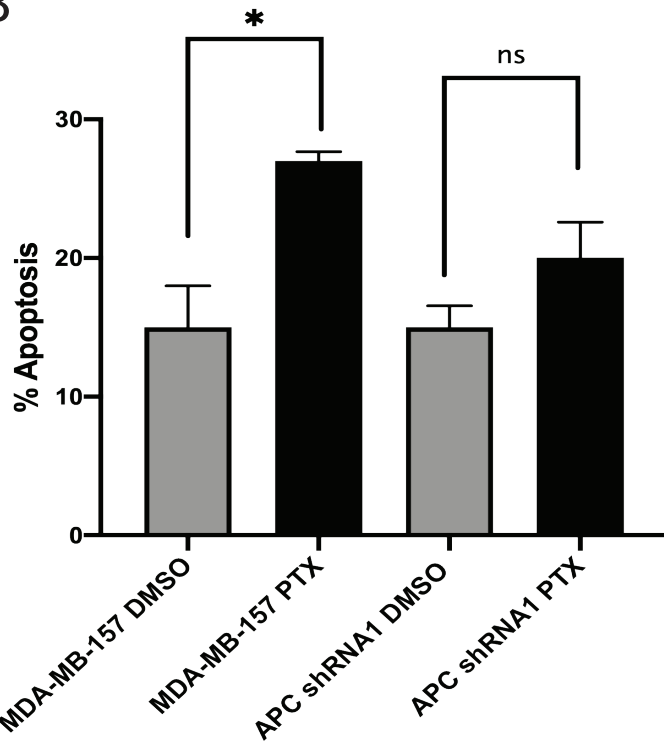

Supplement: S2 Fig — MDA-MB-157 and APC shRNA1 cells were treated with PTX or DMSO control and stained for annexin V and PI. (A) Representative histograms of the apoptotic population in Q2 (late apoptosis) and Q3 (early apoptosis). (B) Quantification of the combined apoptotic population (n = 3). * p < 0.05 comparing PTX to DMSO treated parental MDA-MB-157 cells. No difference was observed after PTX treatment in the APC shRNA1 cells. (PDF) [file pone.0255738.s002.pdf]

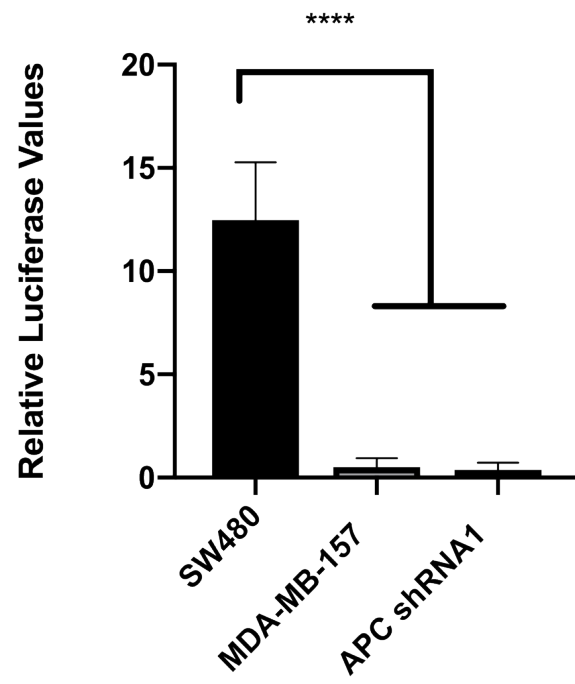

Supplement: S3 Fig — β-catenin/TCF reporter assays showed minimal basal Wnt/β-catenin pathway activation in the APC shRNA1 cells compared to the parental MDA-MB-157. SW480 cells were used as a positive control. The data are shown as a ratio of normalized TOP-Flash values. **** p < 0.0001 compared to SW480 control cells. (PDF) [file pone.0255738.s003.pdf]
