# Supplementary material for: Adenomatous Polyposis Coli loss controls cell cycle regulators and response to paclitaxel in MDA-MB-157 metaplastic breast cancer cells
Source: PLoS One. 2021 Aug 9;16(8):e0255738. doi: 10.1371/journal.pone.0255738 (PMC8351968; doi:10.1371/journal.pone.0255738)
Supplement: S4 Fig — (A) Hierarchical clustering and heat map of the genes associated with the over-represented biological process ontologies of cell cycle/cell division. Input data are the normalized expression values. The values in blue are upregulated and those in red are down-regulated. (B) The description of each gene in the clusters are available from the associated excel file. (PDF) [file pone.0255738.s004.pdf]

and Jennifer R. Prosperi

Supplementary Figure 1A. Cell cycle

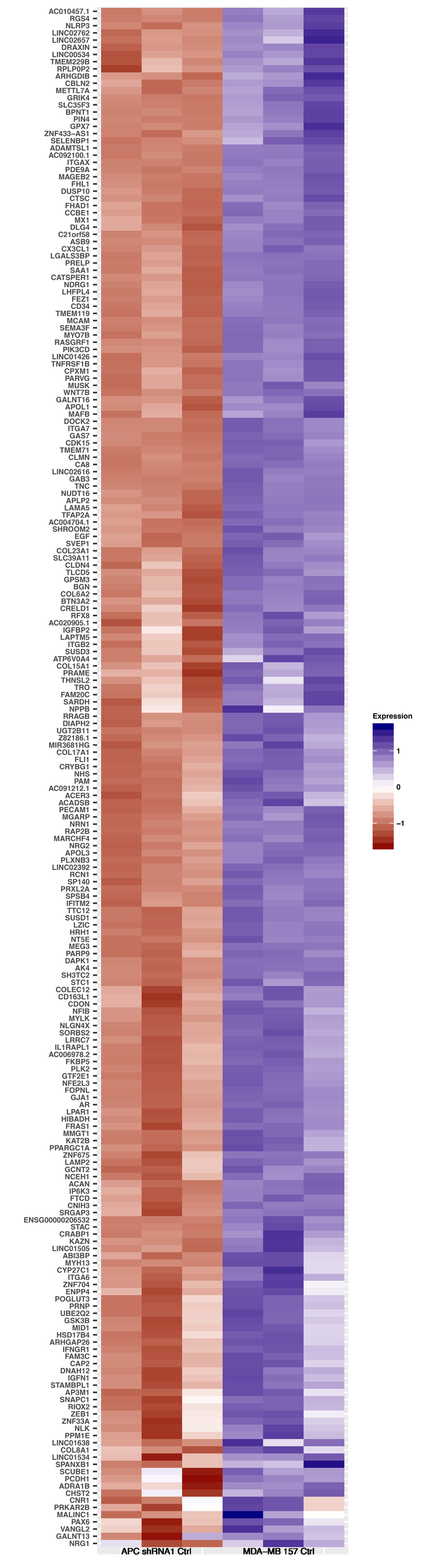

Supplemental Figure 1B. Down-regulated in APC shRNA1 cells

ensembl_gene_id	hgnc	entrezgene_id	clusterOrder	baseMean	log2FoldChange	lfcSE	stat	pvalue	description
ENSG00000251257	AC010457.1 (novel transcript)	NA	214	46.40621	-4.140618	0.5478044	-7.55857	4.08E-14	LncRNA
ENSG00000117152	RGS4	5999	213	2.886760776	-5.961099825	1.354142335	-4.402122044	1.07E-05	regulator of G protein signaling 4 [Source:HGNC Symbol;Acc:HGNC:10000]
ENSG00000162711	NLRP3	114548	212	26.33632458	-2.932799097	0.4072063	-7.202243909	5.92E-13	NLR family pyrin domain containing 3 [Source:HGNC Symbol;Acc:HGNC:16400]
ENSG00000250303	LINC02762	NA	211	45.7454375	-1.587040232	0.266763927	-5.949231033	2.69E-09	long intergenic non-protein coding RNA 2762 [Source:HGNC Symbol;Acc:HGNC:27443]
ENSG00000242147	LINC02657	NA	210	83.16373373	-1.391442155	0.258073612	-5.391648317	6.98E-08	long intergenic non-protein coding RNA 2657 [Source:HGNC Symbol;Acc:HGNC:54143]
ENSG00000162490	DRAXIN	374946	209	29.47874841	-2.497354223	0.352701197	-7.080651397	1.43E-12	dorsal inhibitory axon guidance protein [Source:HGNC Symbol;Acc:HGNC:25054]
ENSG00000253394	LINC00534	NA	208	16.25941805	-0.925089188	0.315367313	-2.933370548	0.003353034	long intergenic non-protein coding RNA 534 [Source:HGNC Symbol;Acc:HGNC:43643]
ENSG00000198133	TMEM229B	161145	207	35.28219165	-1.325904817	0.297233501	-4.460818894	8.16E-06	transmembrane protein 229B [Source:HGNC Symbol;Acc:HGNC:20130]
ENSG00000243742	RPLP0P2	NA	206	26.71983697	-2.15901419	0.371987804	-5.803991877	6.48E-09	ribosomal protein lateral stalk subunit P0 pseudogene 2 [Source:HGNC Symbol;Acc:HGNC:17960]
ENSG00000111348	ARHGDIB	397	205	121.6175867	-1.625309897	0.208135999	-7.808884108	5.77E-15	Rho GDP dissociation inhibitor beta [Source:HGNC Symbol;Acc:HGNC:679]
ENSG00000141668	CBLN2	147381	204	3.558657039	-3.755870532	0.978170698	-3.839688248	0.000123191	cerebellin 2 precursor [Source:HGNC Symbol;Acc:HGNC:1544]
ENSG00000185432	METTL7A	25840	203	133.7953679	-1.936537482	0.192949807	-10.03648312	1.05E-23	methyltransferase like 7A [Source:HGNC Symbol;Acc:HGNC:24550]
ENSG00000149403	GRIK4	2900	202	65.89392186	-1.283414898	0.177651248	-7.224350584	5.04E-13	glutamate ionotropic receptor kainate type subunit 4 [Source:HGNC Symbol;Acc:HGNC:4582]
ENSG00000183780	SLC35F3	148641	201	341.3943602	-0.796980835	0.123802016	-6.437543296	1.21E-10	solute carrier family 35 member F3 [Source:HGNC Symbol;Acc:HGNC:23616]
ENSG00000162813	BPNT1	10380	200	187.8919417	-1.539891345	0.181974594	-8.462122717	2.63E-17	3'(2'), 5'-bisphosphate nucleotidase 1 [Source:HGNC Symbol;Acc:HGNC:1096]
ENSG00000102309	PIN4	5303	199	322.2198344	-1.103333245	0.20165113	-5.471495472	4.46E-08	peptidylprolyl cis/trans isomerase, NIMA-interacting 4 [Source:HGNC Symbol;Acc:HGNC:8992]
ENSG00000116157	GPX7	2882	198	49.45314476	-1.175592452	0.260992656	-4.504312379	6.66E-06	glutathione peroxidase 7 [Source:HGNC Symbol;Acc:HGNC:4559]
ENSG00000219665	ZNF433-AS1	NA	197	76.16161272	-1.076477976	0.197225705	-5.458101815	4.81E-08	ZNF433 and ZNF878 antisense RNA 1 [Source:HGNC Symbol;Acc:HGNC:53776]
ENSG00000143416	SELENBP1	8991	196	9.174928441	-5.626291698	1.251351682	-4.496171444	6.92E-06	selenium binding protein 1 [Source:HGNC Symbol;Acc:HGNC:10719]
ENSG00000178031	ADAMTSL1	92949	195	59.12260065	-7.11352469	0.862432513	-8.248210242	1.61E-16	ADAMTS like 1 [Source:HGNC Symbol;Acc:HGNC:14632]
ENSG00000140678	ITGAX	3687	193	25.46423394	-3.504827331	0.440154449	-7.962721588	1.68E-15	integrin subunit alpha X [Source:HGNC Symbol;Acc:HGNC:6152]
ENSG00000160191	PDE9A	5152	192	285.1329577	-1.886132674	0.136528631	-13.8149241	2.07E-43	phosphodiesterase 9A [Source:HGNC Symbol;Acc:HGNC:8795]
ENSG00000099399	MAGEB2	4113	191	52.5643312	-3.151568888	0.301584642	-10.4500311	1.46E-25	MAGE family member B2 [Source:HGNC Symbol;Acc:HGNC:6809]
ENSG00000022267	FHL1	2273	190	489.7351177	-1.269307148	0.115634867	-10.97685485	4.94E-28	four and a half LIM domains 1 [Source:HGNC Symbol;Acc:HGNC:3702]
ENSG00000143507	DUSP10	11221	189	258.4561519	-0.699514715	0.109561133	-6.384697681	1.72E-10	dual specificity phosphatase 10 [Source:HGNC Symbol;Acc:HGNC:3065]
ENSG00000109861	CTSC	1075	188	382.2021163	-1.132987066	0.128797624	-8.796645729	1.41E-18	cathepsin C [Source:HGNC Symbol;Acc:HGNC:2528]
ENSG00000142621	FHAD1	114827	187	229.9370178	-0.829532822	0.124404787	-6.668013692	2.59E-11	forkhead associated phosphopeptide binding domain 1 [Source:HGNC Symbol;Acc:HGNC:29408]
ENSG00000183287	CCBE1	147372	186	143.4809057	-1.260743434	0.180973123	-6.966467787	3.25E-12	collagen and calcium binding EGF domains 1 [Source:HGNC Symbol;Acc:HGNC:29426]
ENSG00000157601	MX1	4599	185	204.2734451	-2.397744503	0.191283062	-12.53505917	4.8E-36	MX dynamin like GTPase 1 [Source:HGNC Symbol;Acc:HGNC:7532]
ENSG00000132535	DLG4	1742	184	217.0253589	-1.26497935	0.160048831	-7.903708787	2.71E-15	discs large MAGUK scaffold protein 4 [Source:HGNC Symbol;Acc:HGNC:2903]
ENSG00000160298	C21orf58	54058	183	190.484774	-1.621936805	0.174106088	-9.315796039	1.21E-20	chromosome 21 open reading frame 58 [Source:HGNC Symbol;Acc:HGNC:1300]
ENSG00000102048	ASB9	140462	182	206.9285738	-1.423313657	0.139558117	-10.19871638	2.01E-24	ankyrin repeat and SOCS box containing 9 [Source:HGNC Symbol;Acc:HGNC:17184]
ENSG00000006210	CX3CL1	6376	181	35.29575466	-2.617326968	0.385066861	-6.797071443	1.07E-11	C-X3-C motif chemokine ligand 1 [Source:HGNC Symbol;Acc:HGNC:10647]
ENSG00000108679	LGALS3BP	3959	180	706.0587837	-2.090293226	0.15977226	-13.08295462	4.12E-39	galectin 3 binding protein [Source:HGNC Symbol;Acc:HGNC:6564]
ENSG00000188783	PRELP	5549	179	19.08676785	-4.068746444	0.58206965	-6.990136732	2.75E-12	proline and arginine rich end leucine rich repeat protein [Source:HGNC Symbol;Acc:HGNC:9357]
ENSG00000173432	SAA1	6288	178	78.11809159	-2.758263738	0.238364289	-11.57163158	5.74E-31	serum amyloid A1 [Source:HGNC Symbol;Acc:HGNC:10513]
ENSG00000175294	CATSPER1	117144	177	65.6726391	-1.763438603	0.24134308	-7.306770952	2.74E-13	cation channel sperm associated 1 [Source:HGNC Symbol;Acc:HGNC:17116]
ENSG00000104419	NDRG1	10397	176	258.5915067	-1.468110999	0.156533278	-9.378906633	6.67E-21	N-myc downstream regulated 1 [Source:HGNC Symbol;Acc:HGNC:7679]
ENSG00000156959	LHFPL4	375323	175	92.52986166	-1.507573635	0.229391036	-6.57206864	4.96E-11	LHFPL tetraspan subfamily member 4 [Source:HGNC Symbol;Acc:HGNC:29568]
ENSG00000149557	FEZ1	9638	174	79.86238906	-2.180021408	0.217570843	-10.01982333	1.25E-23	fasciculation and elongation protein zeta 1 [Source:HGNC Symbol;Acc:HGNC:3659]
ENSG00000174059	CD34	947	173	76.94189246	-1.578200028	0.206912845	-7.627366146	2.4E-14	CD34 molecule [Source:HGNC Symbol;Acc:HGNC:1662]
ENSG00000183160	TMEM119	338773	172	251.328967	-3.194345813	0.296937093	-10.75765167	5.45E-27	transmembrane protein 119 [Source:HGNC Symbol;Acc:HGNC:27884]
ENSG00000076706	MCAM	4162	171	1433.917112	-2.387346936	0.136205413	-17.52754816	8.83E-69	melanoma cell adhesion molecule [Source:HGNC Symbol;Acc:HGNC:6934]
ENSG00000001617	SEMA3F	6405	170	70.9042249	-2.296808594	0.300952134	-7.631806972	2.31E-14	semaphorin 3F [Source:HGNC Symbol;Acc:HGNC:10728]
ENSG00000169994	MYO7B	4648	169	468.6569745	-1.457460149	0.160118109	-9.102406675	8.84E-20	myosin VIIB [Source:HGNC Symbol;Acc:HGNC:7607]
ENSG00000058335	RASGRF1	5923	168	29.76431607	-3.708251296	0.387555624	-9.56830727	1.09E-21	Ras protein specific guanine nucleotide releasing factor 1 [Source:HGNC Symbol;Acc:HGNC:9875]
ENSG00000171608	PIK3CD	5293	167	1291.971431	-1.068394799	0.121689058	-8.779711283	1.64E-18	phosphatidylinositol-4,5-bisphosphate 3-kinase catalytic subunit delta [Source:HGNC Symbol;Acc:HGNC:8977]
ENSG00000234380	LINC01426	NA	166	37.30933079	-3.453893089	0.38875642	-8.884465715	6.42E-19	long intergenic non-protein coding RNA 1426 [Source:HGNC Symbol;Acc:HGNC:50734]
ENSG00000028137	TNFRSF1B	7133	165	100.4108151	-2.989002991	0.269420375	-11.09419802	1.34E-28	TNF receptor superfamily member 1B [Source:HGNC Symbol;Acc:HGNC:11917]
ENSG00000088882	CPXM1	56265	164	268.2518574	-2.278419746	0.184177661	-12.37077142	3.76E-35	carboxypeptidase X, M14 family member 1 [Source:HGNC Symbol;Acc:HGNC:15771]
ENSG00000138964	PARVG	64098	163	8.432547075	-5.008193835	1.096203784	-4.56867045	4.91E-06	parvin gamma [Source:HGNC Symbol;Acc:HGNC:14654]
ENSG00000030304	MUSK	4593	162	40.66025609	-1.608307237	0.243759297	-6.597931888	4.17E-11	muscle associated receptor tyrosine kinase [Source:HGNC Symbol;Acc:HGNC:7525]
ENSG00000188064	WNT7B	7477	161	283.4068655	-1.903170809	0.198404032	-9.592399852	8.61E-22	Wnt family member 7B [Source:HGNC Symbol;Acc:HGNC:12787]
ENSG00000100626	GALNT16	57452	160	92.03483689	-1.820202684	0.196857056	-9.246316709	2.32E-20	polypeptide N-acetylgalactosaminyltransferase 16 [Source:HGNC Symbol;Acc:HGNC:23233]
ENSG00000100342	APOL1	8542	159	259.7144456	-1.083959747	0.156535791	-6.924676713	4.37E-12	apolipoprotein L1 [Source:HGNC Symbol;Acc:HGNC:618]
ENSG00000204103	MAFB	9935	158	16.98626028	-2.077646575	0.411479789	-5.049206866	4.44E-07	MAF bZIP transcription factor B [Source:HGNC Symbol;Acc:HGNC:6408]
ENSG00000134516	DOCK2	1794	157	82.6388376	-1.012308456	0.152985391	-6.617026977	3.66E-11	dedicator of cytokinesis 2 [Source:HGNC Symbol;Acc:HGNC:2988]
ENSG00000135424	ITGA7	3679	156	143.3263873	-1.449222037	0.18298235	-7.920009965	2.37E-15	integrin subunit alpha 7 [Source:HGNC Symbol;Acc:HGNC:6143]
ENSG00000007237	GAS7	8522	155	340.4038808	-1.417037332	0.126081721	-11.23903859	2.62E-29	growth arrest specific 7 [Source:HGNC Symbol;Acc:HGNC:4169]
ENSG00000138395	CDK15	65061	154	44.75306801	-4.345568655	0.442805352	-9.813722059	9.83E-23	cyclin dependent kinase 15 [Source:HGNC Symbol;Acc:HGNC:14434]
ENSG00000165071	TMEM71	137835	153	3.711185495	-5.916570383	1.291968706	-4.579499763	4.66E-06	transmembrane protein 71 [Source:HGNC Symbol;Acc:HGNC:26572]
ENSG00000165959	CLMN	79789	152	88.26540724	-1.681952596	0.176183093	-9.546617509	1.34E-21	calmin [Source:HGNC Symbol;Acc:HGNC:19972]
ENSG00000178538	CA8	767	151	240.2527914	-3.397358874	0.154508548	-21.9881613	3.74E-107	carbonic anhydrase 8 [Source:HGNC Symbol;Acc:HGNC:1382]
ENSG00000261761	LINC02616	NA	150	7.11447832	-7.614418372	1.302292991	-5.846931855	5.01E-09	long intergenic non-protein coding RNA 2616 [Source:HGNC Symbol;Acc:HGNC:54078]
ENSG00000160219	GAB3	139716	149	35.14442464	-1.931096649	0.22712901	-8.502201659	1.86E-17	GRB2 associated binding protein 3 [Source:HGNC Symbol;Acc:HGNC:17515]
ENSG00000041982	TNC	3371	148	1038.988883	-1.192705673	0.179708131	-6.636904329	3.2E-11	tenascin C [Source:HGNC Symbol;Acc:HGNC:5318]
ENSG00000198585	NUDT16	131870	147	268.064243	-0.911354014	0.122830185	-7.419625844	1.17E-13	nudix hydrolase 16 [Source:HGNC Symbol;Acc:HGNC:26442]
ENSG00000084234	APLP2	334	146	1317.181898	-1.240670038	0.142871989	-8.683787828	3.83E-18	amyloid beta precursor like protein 2 [Source:HGNC Symbol;Acc:HGNC:598]
ENSG00000130702	LAMA5	3911	145	814.5235712	-0.633889969	0.149450432	-4.241472987	2.22E-05	laminin subunit alpha 5 [Source:HGNC Symbol;Acc:HGNC:6485]
ENSG00000137203	TFAP2A	7020	144	173.0363684	-1.184576862	0.129371472	-9.156399368	5.37E-20	transcription factor AP-2 alpha [Source:HGNC Symbol;Acc:HGNC:11742]
ENSG00000146950	SHROOM2	357	142	294.1338521	-0.935671649	0.134189404	-6.972768511	3.11E-12	shroom family member 2 [Source:HGNC Symbol;Acc:HGNC:630]
ENSG00000138798	EGF	1950	141	14.66570779	-3.725145139	0.515415048	-7.22746678	4.92E-13	epidermal growth factor [Source:HGNC Symbol;Acc:HGNC:3229]
ENSG00000165124	SVEP1	79987	140	42.65244567	-1.97576714	0.238205148	-8.29439313	1.09E-16	sushi, von Willebrand factor type A, EGF and pentraxin domain containing 1 [Source:HGNC Symbol;Acc:HGNC:1
ENSG00000050767	COL23A1	91522	139	59.82289889	-1.905216862	0.239451744	-7.956579591	1.77E-15	collagen type XXIII alpha 1 chain [Source:HGNC Symbol;Acc:HGNC:22990]
ENSG00000133195	SLC39A11	201266	138	333.0534858	-0.699805759	0.10162273	-6.886311322	5.73E-12	solute carrier family 39 member 11 [Source:HGNC Symbol;Acc:HGNC:14463]
ENSG00000189143	CLDN4	1364	137	102.7408441	-1.154944526	0.189845111	-6.0836148	1.18E-09	claudin 4 [Source:HGNC Symbol;Acc:HGNC:2046]
ENSG00000181264	TLCD5	219902	136	111.5915889	-1.017560765	0.151835472	-6.701732818	2.06E-11	TLC domain containing 5 [Source:HGNC Symbol;Acc:HGNC:28280]
ENSG00000213654	GPSM3	63940	135	228.1032992	-1.122640731	0.207099034	-5.420791729	5.93E-08	G protein signaling modulator 3 [Source:HGNC Symbol;Acc:HGNC:13945]
ENSG00000182492	BGN	633	134	1221.216004	-1.324471335	0.1897822	-6.978901784	2.97E-12	biglycan [Source:HGNC Symbol;Acc:HGNC:1044]
ENSG00000142173	COL6A2	1292	133	6948.413222	-1.076581646	0.168634493	-6.384112922	1.72E-10	collagen type VI alpha 2 chain [Source:HGNC Symbol;Acc:HGNC:2212]
ENSG00000186470	BTN3A2	11118	132	205.5227998	-1.220302487	0.144327422	-8.455097962	2.79E-17	butyrophilin subfamily 3 member A2 [Source:HGNC Symbol;Acc:HGNC:1139]
ENSG00000163703	CRELD1	78987	131	263.6915069	-1.014731317	0.176735127	-5.741537268	9.38E-09	cysteine rich with EGF like domains 1 [Source:HGNC Symbol;Acc:HGNC:14630]
ENSG00000196460	RFX8	731220	130	17.88140664	-2.545766339	0.575124235	-4.426463335	9.58E-06	RFX family member 8, lacking RFX DNA binding domain [Source:HGNC Symbol;Acc:HGNC:37253]
ENSG00000115457	IGFBP2	3485	128	934.7831821	-0.924911192	0.219161635	-4.220223995	2.44E-05	insulin like growth factor binding protein 2 [Source:HGNC Symbol;Acc:HGNC:5471]
ENSG00000162511	LAPTM5	7805	127	61.9029448	-1.950137203	0.241000072	-8.091853195	5.88E-16	lysosomal protein transmembrane 5 [Source:HGNC Symbol;Acc:HGNC:29612]
ENSG00000160255	ITGB2	3689	126	171.7893614	-1.18962744	0.159796576	-7.444636598	9.72E-14	integrin subunit beta 2 [Source:HGNC Symbol;Acc:HGNC:6155]
ENSG00000157303	SUSD3	203328	125	170.8680292	-0.742091788	0.197685719	-3.753896797	0.000174107	sushi domain containing 3 [Source:HGNC Symbol;Acc:HGNC:28391]
ENSG00000105929	ATP6V0A4	50617	124	29.57627406	-0.762903563	0.238381002	-3.20035387	0.00137259	ATPase H+ transporting V0 subunit a4 [Source:HGNC Symbol;Acc:HGNC:866]
ENSG00000204291	COL15A1	1306	123	44.19679194	-1.215527413	0.301587906	-4.030424921	5.57E-05	collagen type XV alpha 1 chain [Source:HGNC Symbol;Acc:HGNC:2192]
ENSG00000185686	PRAME	23532	122	360.8696045	-0.890076266	0.127313523	-6.991215423	2.73E-12	preferentially expressed antigen in melanoma [Source:HGNC Symbol;Acc:HGNC:9336]
ENSG00000144115	THNSL2	55258	121	21.44919615	-1.030303327	0.347052363	-2.968725865	0.002990372	threonine synthase like 2 [Source:HGNC Symbol;Acc:HGNC:25602]
ENSG00000067445	TRO	7216	120	217.9025489	-0.78170635	0.152154428	-5.137585283	2.78E-07	trophinin [Source:HGNC Symbol;Acc:HGNC:12326]
ENSG00000177706	FAM20C	56975	119	2663.376615	-0.793906688	0.146293653	-5.426801999	5.74E-08	FAM20C golgi associated secretory pathway kinase [Source:HGNC Symbol;Acc:HGNC:22140]
ENSG00000123453	SARDH	1757	118	90.24681971	-0.732595086	0.187232217	-3.912761909	9.12E-05	sarcosine dehydrogenase [Source:HGNC Symbol;Acc:HGNC:10536]
ENSG00000120937	NPPB	4879	117	7.452570779	-1.191961402	0.598997449	-1.989927344	0.04659894	natriuretic peptide B [Source:HGNC Symbol;Acc:HGNC:7940]
ENSG00000083750	RRAGB	10325	116	35.22183348	-1.110096424	0.226640139	-4.898057455	9.68E-07	Ras related GTP binding B [Source:HGNC Symbol;Acc:HGNC:19901]
ENSG00000147202	DIAPH2	1730	115	6.334784452	-3.486423372	0.722829321	-4.823300982	1.41E-06	diaphanous related formin 2 [Source:HGNC Symbol;Acc:HGNC:2877]
ENSG00000213759	UGT2B11	10720	114	17.21461076	-3.059284201	0.446873543	-6.845972988	7.6E-12	UDP glucuronosyltransferase family 2 member B11 [Source:HGNC Symbol;Acc:HGNC:12545]
ENSG00000224184	MIR3681HG	NA	112	9.590563	-3.151834932	0.584327861	-5.393949431	6.89E-08	MIR3681 host gene [Source:HGNC Symbol;Acc:HGNC:52001]
ENSG00000065618	COL17A1	1308	111	18.51926349	-3.895064863	0.600335411	-6.488147776	8.69E-11	collagen type XVII alpha 1 chain [Source:HGNC Symbol;Acc:HGNC:2194]
ENSG00000151702	FLI1	2313	110	132.8982471	-1.340029211	0.156285972	-8.574212976	9.98E-18	Fli-1 proto-oncogene, ETS transcription factor [Source:HGNC Symbol;Acc:HGNC:3749]
ENSG00000112297	CRYBG1	202	109	123.2291647	-1.587289858	0.196718603	-8.068834551	7.1E-16	crystallin beta-gamma domain containing 1 [Source:HGNC Symbol;Acc:HGNC:356]
ENSG00000188158	NHS	4810	108	9.986941469	-2.975375438	0.493659765	-6.027178328	1.67E-09	NHS actin remodeling regulator [Source:HGNC Symbol;Acc:HGNC:7820]
ENSG00000145730	PAM	5066	107	366.8405667	-0.831921034	0.141785063	-5.867480079	4.42E-09	peptidylglycine alpha-amidating monooxygenase [Source:HGNC Symbol;Acc:HGNC:8596]
ENSG00000078124	ACER3	55331	105	211.0907109	-0.850168497	0.155452647	-5.468986961	4.53E-08	alkaline ceramidase 3 [Source:HGNC Symbol;Acc:HGNC:16066]
ENSG00000196177	ACADSB	36	104	64.93959837	-1.184906488	0.219638925	-5.394792796	6.86E-08	acyl-CoA dehydrogenase short/branched chain [Source:HGNC Symbol;Acc:HGNC:91]
ENSG00000261371	PECAM1	5175	103	17.91889676	-4.296645414	0.644477895	-6.66686235	2.61E-11	platelet and endothelial cell adhesion molecule 1 [Source:HGNC Symbol;Acc:HGNC:8823]
ENSG00000137463	MGARP	84709	102	43.01939235	-0.960617798	0.239284497	-4.014542558	5.96E-05	mitochondria localized glutamic acid rich protein [Source:HGNC Symbol;Acc:HGNC:29969]
ENSG00000124785	NRN1	51299	101	73.68785386	-2.520073462	0.250848488	-10.04619753	9.55E-24	neuritin 1 [Source:HGNC Symbol;Acc:HGNC:17972]
ENSG00000181467	RAP2B	5912	100	134.8004641	-0.621464118	0.137636618	-4.515252757	6.32E-06	RAP2B, member of RAS oncogene family [Source:HGNC Symbol;Acc:HGNC:9862]
ENSG00000144583	MARCHF4	57574	99	39.40796104	-1.765889089	0.248058681	-7.118836069	1.09E-12	membrane associated ring-CH-type finger 4 [Source:HGNC Symbol;Acc:HGNC:29269]
ENSG00000158458	NRG2	9542	98	31.70234636	-2.857348394	0.353331685	-8.086872793	6.12E-16	neuregulin 2 [Source:HGNC Symbol;Acc:HGNC:7998]
ENSG00000128284	APOL3	80833	97	58.37850508	-2.315042472	0.214198791	-10.80791571	3.16E-27	apolipoprotein L3 [Source:HGNC Symbol;Acc:HGNC:14868]
ENSG00000198753	PLXNB3	5365	96	35.6845025	-1.718167841	0.316324602	-5.431660486	5.58E-08	plexin B3 [Source:HGNC Symbol;Acc:HGNC:9105]
ENSG00000258183	LINC02392	NA	95	59.10712565	-2.671478275	0.252078107	-10.59781949	3.05E-26	long intergenic non-protein coding RNA 2392 [Source:HGNC Symbol;Acc:HGNC:53319]
ENSG00000049449	RCN1	5954	94	125.5211653	-1.997813405	0.173926449	-11.48654171	1.54E-30	reticulocalbin 1 [Source:HGNC Symbol;Acc:HGNC:9934]
ENSG00000079263	SP140	11262	93	68.51547527	-2.614313886	0.232018918	-11.26767553	1.9E-29	SP140 nuclear body protein [Source:HGNC Symbol;Acc:HGNC:17133]
ENSG00000122378	PRXL2A	84293	92	71.52115588	-2.232623729	0.198772663	-11.23204615	2.84E-29	peroxiredoxin like 2A [Source:HGNC Symbol;Acc:HGNC:28651]
ENSG00000175093	SPSB4	92369	91	22.25460831	-1.75427003	0.32007485	-5.480811846	4.23E-08	splA/ryanodine receptor domain and SOCS box containing 4 [Source:HGNC Symbol;Acc:HGNC:30630]
ENSG00000185201	IFITM2	10581	90	130.7381763	-1.982128502	0.200943216	-9.864122537	5.96E-23	interferon induced transmembrane protein 2 [Source:HGNC Symbol;Acc:HGNC:5413]
ENSG00000149292	TTC12	54970	89	32.49033132	-2.413649476	0.292115075	-8.262666615	1.42E-16	tetratricopeptide repeat domain 12 [Source:HGNC Symbol;Acc:HGNC:23700]
ENSG00000106868	SUSD1	64420	88	127.4509845	-0.742722749	0.135776961	-5.470167728	4.5E-08	sushi domain containing 1 [Source:HGNC Symbol;Acc:HGNC:25413]
ENSG00000162441	LZIC	84328	87	301.2743977	-0.850020682	0.114821215	-7.402993292	1.33E-13	leucine zipper and CTNNBIP1 domain containing [Source:HGNC Symbol;Acc:HGNC:17497]
ENSG00000196639	HRH1	3269	86	350.7500031	-0.760981988	0.094393258	-8.061825619	7.52E-16	histamine receptor H1 [Source:HGNC Symbol;Acc:HGNC:5182]
ENSG00000135318	NT5E	4907	85	377.2716698	-1.51211786	0.122943193	-12.29932156	9.13E-35	5'-nucleotidase ecto [Source:HGNC Symbol;Acc:HGNC:8021]
ENSG00000214548	MEG3	NA	84	14.0777437	-4.338749702	0.578124896	-7.504865694	6.15E-14	maternally expressed 3 [Source:HGNC Symbol;Acc:HGNC:14575]
ENSG00000138496	PARP9	83666	83	74.46171713	-1.224406172	0.183572431	-6.669880462	2.56E-11	poly(ADP-ribose) polymerase family member 9 [Source:HGNC Symbol;Acc:HGNC:24118]
ENSG00000196730	DAPK1	1612	82	42.73672522	-1.841939181	0.236455287	-7.789799093	6.71E-15	death associated protein kinase 1 [Source:HGNC Symbol;Acc:HGNC:2674]
ENSG00000162433	AK4	205	81	117.9357781	-1.204263949	0.13927529	-8.646644688	5.3E-18	adenylate kinase 4 [Source:HGNC Symbol;Acc:HGNC:363]
ENSG00000169247	SH3TC2	79628	80	170.5441139	-0.741918656	0.119332604	-6.217233455	5.06E-10	SH3 domain and tetratricopeptide repeats 2 [Source:HGNC Symbol;Acc:HGNC:29427]
ENSG00000159167	STC1	6781	79	89.50391616	-1.271845747	0.167602899	-7.588447186	3.24E-14	stanniocalcin 1 [Source:HGNC Symbol;Acc:HGNC:11373]
ENSG00000158270	COLEC12	81035	78	334.0143966	-0.983424202	0.142767813	-6.88827673	5.65E-12	collectin subfamily member 12 [Source:HGNC Symbol;Acc:HGNC:16016]
ENSG00000177675	CD163L1	283316	77	72.29687436	-1.321107213	0.241950459	-5.460238511	4.75E-08	CD163 molecule like 1 [Source:HGNC Symbol;Acc:HGNC:30375]
ENSG00000064309	CDON	50937	76	34.61523189	-1.981680266	0.268799476	-7.37233679	1.68E-13	cell adhesion associated, oncogene regulated [Source:HGNC Symbol;Acc:HGNC:17104]
ENSG00000147862	NFIB	4781	75	122.5089365	-1.326094856	0.206323419	-6.427262892	1.3E-10	nuclear factor I B [Source:HGNC Symbol;Acc:HGNC:7785]
ENSG00000065534	MYLK	4638	74	221.7420353	-1.403273906	0.148504262	-9.44938472	3.41E-21	myosin light chain kinase [Source:HGNC Symbol;Acc:HGNC:7590]
ENSG00000146938	NLGN4X	57502	73	282.0650059	-1.531307973	0.167649885	-9.133963744	6.6E-20	neuroligin 4 X-linked [Source:HGNC Symbol;Acc:HGNC:14287]
ENSG00000154556	SORBS2	8470	72	155.0597443	-1.470338932	0.180507533	-8.14558211	3.77E-16	sorbin and SH3 domain containing 2 [Source:HGNC Symbol;Acc:HGNC:24098]
ENSG00000033122	LRRC7	57554	71	23.49335009	-2.110920833	0.304894317	-6.923450899	4.41E-12	leucine rich repeat containing 7 [Source:HGNC Symbol;Acc:HGNC:18531]
ENSG00000169306	IL1RAPL1	11141	70	44.05084833	-2.253377652	0.336485175	-6.696811083	2.13E-11	interleukin 1 receptor accessory protein like 1 [Source:HGNC Symbol;Acc:HGNC:5996]
ENSG00000096060	FKBP5	2289	68	88.6937815	-1.366574465	0.170877257	-7.997404044	1.27E-15	FKBP prolyl isomerase 5 [Source:HGNC Symbol;Acc:HGNC:3721]
ENSG00000145632	PLK2	10769	67	603.0619635	-1.267510521	0.127423896	-9.947196394	2.59E-23	polo like kinase 2 [Source:HGNC Symbol;Acc:HGNC:19699]
ENSG00000153767	GTF2E1	2960	66	75.83249082	-1.080448487	0.191896583	-5.630368562	1.8E-08	general transcription factor IIE subunit 1 [Source:HGNC Symbol;Acc:HGNC:4650]
ENSG00000050344	NFE2L3	9603	65	166.2742673	-1.551232282	0.177261207	-8.751109763	2.11E-18	nuclear factor, erythroid 2 like 3 [Source:HGNC Symbol;Acc:HGNC:7783]
ENSG00000133393	FOPNL	123811	64	246.2025805	-0.870954668	0.129058573	-6.748522384	1.49E-11	FGFR1OP N-terminal like [Source:HGNC Symbol;Acc:HGNC:26435]
ENSG00000152661	GJA1	2697	63	133.0112079	-2.391409444	0.1956995	-12.21980357	2.44E-34	gap junction protein alpha 1 [Source:HGNC Symbol;Acc:HGNC:4274]
ENSG00000169083	AR	367	62	34.7777221	-1.710428879	0.235735825	-7.255701935	4E-13	androgen receptor [Source:HGNC Symbol;Acc:HGNC:644]
ENSG00000198121	LPAR1	1902	61	334.5648867	-1.161158062	0.161865688	-7.173589901	7.31E-13	lysophosphatidic acid receptor 1 [Source:HGNC Symbol;Acc:HGNC:3166]
ENSG00000106049	HIBADH	11112	60	159.5481836	-1.389478158	0.206032926	-6.743961687	1.54E-11	3-hydroxyisobutyrate dehydrogenase [Source:HGNC Symbol;Acc:HGNC:4907]
ENSG00000138759	FRAS1	80144	59	41.01780323	-1.689431278	0.215681373	-7.832995746	4.76E-15	Fraser extracellular matrix complex subunit 1 [Source:HGNC Symbol;Acc:HGNC:19185]
ENSG00000169446	MMGT1	93380	58	369.781027	-1.065437166	0.109861408	-9.698011246	3.07E-22	membrane magnesium transporter 1 [Source:HGNC Symbol;Acc:HGNC:28100]
ENSG00000114166	KAT2B	8850	57	67.87638451	-1.823571368	0.258892676	-7.043734876	1.87E-12	lysine acetyltransferase 2B [Source:HGNC Symbol;Acc:HGNC:8638]
ENSG00000109819	PPARGC1A	10891	56	2.969906582	-3.704636152	0.979555434	-3.781956612	0.0001556	PPARG coactivator 1 alpha [Source:HGNC Symbol;Acc:HGNC:9237]
ENSG00000197372	ZNF675	171392	55	335.6105243	-0.881458573	0.123022598	-7.165013465	7.78E-13	zinc finger protein 675 [Source:HGNC Symbol;Acc:HGNC:30768]
ENSG00000005893	LAMP2	3920	54	1437.33728	-0.685113201	0.096758943	-7.080618873	1.44E-12	lysosomal associated membrane protein 2 [Source:HGNC Symbol;Acc:HGNC:6501]
ENSG00000111846	GCNT2	2651	53	166.9527499	-1.254180395	0.165713395	-7.568370646	3.78E-14	glucosaminyl (N-acetyl) transferase 2 (I blood group) [Source:HGNC Symbol;Acc:HGNC:4204]
ENSG00000144959	NCEH1	57552	52	131.4529735	-0.957784539	0.175158834	-5.468091545	4.55E-08	neutral cholesterol ester hydrolase 1 [Source:HGNC Symbol;Acc:HGNC:29260]
ENSG00000157766	ACAN	176	51	11.52482147	-3.081214812	0.515827416	-5.973344407	2.32E-09	aggrecan [Source:HGNC Symbol;Acc:HGNC:319]
ENSG00000161896	IP6K3	117283	50	26.04327393	-1.393116253	0.296635591	-4.696389424	2.65E-06	inositol hexakisphosphate kinase 3 [Source:HGNC Symbol;Acc:HGNC:17269]
ENSG00000160282	FTCD	10841	49	14.42522191	-2.32876975	0.427712141	-5.444712752	5.19E-08	formimidoyltransferase cyclodeaminase [Source:HGNC Symbol;Acc:HGNC:3974]
ENSG00000143786	CNIH3	149111	48	44.62630891	-1.778851312	0.256477289	-6.935706926	4.04E-12	cornichon family AMPA receptor auxiliary protein 3 [Source:HGNC Symbol;Acc:HGNC:26802]
ENSG00000196220	SRGAP3	9901	47	20.12754286	-2.019046283	0.317494092	-6.359319235	2.03E-10	SLIT-ROBO Rho GTPase activating protein 3 [Source:HGNC Symbol;Acc:HGNC:19744]
ENSG00000144681	STAC	6769	45	34.08169804	-1.752973788	0.277241269	-6.322917928	2.57E-10	SH3 and cysteine rich domain [Source:HGNC Symbol;Acc:HGNC:11353]
ENSG00000166426	CRABP1	1381	44	9.151373705	-5.89671295	1.245047428	-4.736135199	2.18E-06	cellular retinoic acid binding protein 1 [Source:HGNC Symbol;Acc:HGNC:2338]
ENSG00000189337	KAZN	23254	43	189.1084341	-0.615342518	0.118444767	-5.195185359	2.05E-07	kazrin, periplakin interacting protein [Source:HGNC Symbol;Acc:HGNC:29173]
ENSG00000234323	LINC01505	NA	42	9.718072006	-3.230669508	0.593982879	-5.438994325	5.36E-08	long intergenic non-protein coding RNA 1505 [Source:HGNC Symbol;Acc:HGNC:51186]
ENSG00000154175	ABI3BP	25890	41	33.28890654	-1.492664478	0.308238627	-4.842561403	1.28E-06	ABI family member 3 binding protein [Source:HGNC Symbol;Acc:HGNC:17265]
ENSG00000006788	MYH13	8735	40	2.868562721	-6.326614776	1.397796135	-4.526135549	6.01E-06	myosin heavy chain 13 [Source:HGNC Symbol;Acc:HGNC:7571]
ENSG00000186684	CYP27C1	339761	39	26.97659291	-0.882714795	0.355851447	-2.480571044	0.013117212	cytochrome P450 family 27 subfamily C member 1 [Source:HGNC Symbol;Acc:HGNC:33480]
ENSG00000091409	ITGA6	3655	38	35.32527278	-1.93492003	0.270153461	-7.16229961	7.93E-13	integrin subunit alpha 6 [Source:HGNC Symbol;Acc:HGNC:6142]
ENSG00000164684	ZNF704	619279	37	57.36866785	-1.057112099	0.21435784	-4.931529909	8.16E-07	zinc finger protein 704 [Source:HGNC Symbol;Acc:HGNC:32291]
ENSG00000001561	ENPP4	22875	36	12.20646505	-1.965304001	0.416323115	-4.720621874	2.35E-06	ectonucleotide pyrophosphatase/phosphodiesterase 4 [Source:HGNC Symbol;Acc:HGNC:3359]
ENSG00000178202	POGLUT3	143888	35	338.6668492	-1.369455071	0.25273212	-5.418603199	6.01E-08	protein O-glucosyltransferase 3 [Source:HGNC Symbol;Acc:HGNC:28496]
ENSG00000171867	PRNP	5621	34	739.6520221	-0.869920406	0.132918116	-6.544784336	5.96E-11	prion protein [Source:HGNC Symbol;Acc:HGNC:9449]
ENSG00000140367	UBE2Q2	92912	33	150.7054772	-1.407005843	0.245117856	-5.740119725	9.46E-09	ubiquitin conjugating enzyme E2 Q2 [Source:HGNC Symbol;Acc:HGNC:19248]
ENSG00000082701	GSK3B	2932	32	443.5888393	-0.59747232	0.14093613	-4.239312658	2.24E-05	glycogen synthase kinase 3 beta [Source:HGNC Symbol;Acc:HGNC:4617]
ENSG00000101871	MID1	4281	31	1020.492372	-0.994334554	0.148837141	-6.680688339	2.38E-11	midline 1 [Source:HGNC Symbol;Acc:HGNC:7095]
ENSG00000133835	HSD17B4	3295	30	326.0065071	-0.677877201	0.138869586	-4.881394268	1.05E-06	hydroxysteroid 17-beta dehydrogenase 4 [Source:HGNC Symbol;Acc:HGNC:5213]
ENSG00000145819	ARHGAP26	23092	29	151.9507233	-0.815464624	0.16721004	-4.876887918	1.08E-06	Rho GTPase activating protein 26 [Source:HGNC Symbol;Acc:HGNC:17073]
ENSG00000027697	IFNGR1	3459	28	189.9437664	-0.920058208	0.170881308	-5.384194561	7.28E-08	interferon gamma receptor 1 [Source:HGNC Symbol;Acc:HGNC:5439]
ENSG00000196937	FAM3C	10447	27	213.4378552	-1.218134078	0.169316792	-7.194407978	6.27E-13	FAM3 metabolism regulating signaling molecule C [Source:HGNC Symbol;Acc:HGNC:18664]
ENSG00000112186	CAP2	10486	26	381.978783	-0.75506647	0.130216337	-5.798554063	6.69E-09	cyclase associated actin cytoskeleton regulatory protein 2 [Source:HGNC Symbol;Acc:HGNC:20039]
ENSG00000174844	DNAH12	201625	25	26.81751841	-2.234096354	0.39372744	-5.674220603	1.39E-08	dynein axonemal heavy chain 12 [Source:HGNC Symbol;Acc:HGNC:2943]
ENSG00000163395	IGFN1	91156	24	111.819379	-1.479215786	0.221479721	-6.678786564	2.41E-11	immunoglobulin like and fibronectin type III domain containing 1 [Source:HGNC Symbol;Acc:HGNC:24607]
ENSG00000138134	STAMBPL1	57559	23	49.14379247	-1.182771447	0.258459659	-4.576232323	4.73E-06	STAM binding protein like 1 [Source:HGNC Symbol;Acc:HGNC:24105]
ENSG00000185009	AP3M1	26985	22	191.4552484	-1.149119484	0.243937399	-4.710714665	2.47E-06	adaptor related protein complex 3 subunit mu 1 [Source:HGNC Symbol;Acc:HGNC:569]
ENSG00000023608	SNAPC1	6617	21	57.06277954	-0.665667927	0.299753131	-2.220720511	0.026369897	small nuclear RNA activating complex polypeptide 1 [Source:HGNC Symbol;Acc:HGNC:11134]
ENSG00000170854	RIOX2	84864	20	129.1073237	-0.806889276	0.199116974	-4.052337991	5.07E-05	ribosomal oxygenase 2 [Source:HGNC Symbol;Acc:HGNC:19441]
ENSG00000148516	ZEB1	6935	19	69.25908653	-1.489476472	0.342373474	-4.350443554	1.36E-05	zinc finger E-box binding homeobox 1 [Source:HGNC Symbol;Acc:HGNC:11642]
ENSG00000189180	ZNF33A	7581	18	88.8758097	-1.315869474	0.242556866	-5.424993707	5.8E-08	zinc finger protein 33A [Source:HGNC Symbol;Acc:HGNC:13096]
ENSG00000087095	NLK	51701	17	90.14039085	-0.947139304	0.19203565	-4.932101443	8.13E-07	nemo like kinase [Source:HGNC Symbol;Acc:HGNC:29858]
ENSG00000175175	PPM1E	22843	16	43.4090471	-0.755109362	0.234568852	-3.21913739	0.001285769	protein phosphatase, Mg2+/Mn2+ dependent 1E [Source:HGNC Symbol;Acc:HGNC:19322]
ENSG00000233521	LINC01638	105372978	15	10.90758268	-1.281684763	0.449492042	-2.851407019	0.004352621	long intergenic non-protein coding RNA 1638 [Source:HGNC Symbol;Acc:HGNC:52425]
ENSG00000144810	COL8A1	1295	14	54.90580421	-0.588464392	0.202784134	-2.90192522	0.003708771	collagen type VIII alpha 1 chain [Source:HGNC Symbol;Acc:HGNC:2215]
ENSG00000225975	LINC01534	NA	13	39.14756271	-0.77519099	0.235222012	-3.29557164	0.000982217	long intergenic non-protein coding RNA 1534 [Source:HGNC Symbol;Acc:HGNC:51281]
ENSG00000227234	SPANXB1	728695	12	77.23024801	-0.776982145	0.252120292	-3.081791396	0.00205759	SPANX family member B1 [Source:HGNC Symbol;Acc:HGNC:14329]
ENSG00000159307	SCUBE1	80274	11	57.73300288	-0.681191835	0.333467529	-2.04275312	0.041076883	signal peptide, CUB domain and EGF like domain containing 1 [Source:HGNC Symbol;Acc:HGNC:13441]
ENSG00000156453	PCDH1	5097	10	48.93153738	-0.734529771	0.272001756	-2.700459671	0.006924373	protocadherin 1 [Source:HGNC Symbol;Acc:HGNC:8655]
ENSG00000170214	ADRA1B	147	9	60.42023195	-1.108083814	0.232184408	-4.772429917	1.82E-06	adrenoceptor alpha 1B [Source:HGNC Symbol;Acc:HGNC:278]
ENSG00000175040	CHST2	9435	8	106.3545515	-0.695439738	0.191770616	-3.626414467	0.000287384	carbohydrate sulfotransferase 2 [Source:HGNC Symbol;Acc:HGNC:1970]
ENSG00000118432	CNR1	1268	7	5.294011445	-1.445792029	0.589069032	-2.454367741	0.014113257	cannabinoid receptor 1 [Source:HGNC Symbol;Acc:HGNC:2159]
ENSG00000005249	PRKAR2B	5577	6	37.71235692	-0.728825768	0.285196972	-2.555517203	0.010603012	protein kinase cAMP-dependent type II regulatory subunit beta [Source:HGNC Symbol;Acc:HGNC:9392]
ENSG00000245146	MALINC1	NA	5	13.08402792	-1.184357832	0.387600141	-3.055617651	0.002245975	mitosis associated long intergenic non-coding RNA 1 [Source:HGNC Symbol;Acc:HGNC:49009]
ENSG00000007372	PAX6	5080	4	30.35991131	-0.653146491	0.241569151	-2.703766141	0.006855852	paired box 6 [Source:HGNC Symbol;Acc:HGNC:8620]
ENSG00000162738	VANGL2	57216	3	38.98412356	-1.079815539	0.272328243	-3.965125053	7.34E-05	VANGL planar cell polarity protein 2 [Source:HGNC Symbol;Acc:HGNC:15511]
ENSG00000144278	GALNT13	114805	2	33.16780325	-0.662831463	0.270645742	-2.449074051	0.0143224	polypeptide N-acetylgalactosaminyltransferase 13 [Source:HGNC Symbol;Acc:HGNC:23242]
ENSG00000157168	NRG1	3084	1	9.912399205	-0.983091402	0.46764951	-2.102197014	0.035536021	neuregulin 1 [Source:HGNC Symbol;Acc:HGNC:7997]
