# Supplementary material for: Adenomatous Polyposis Coli loss controls cell cycle regulators and response to paclitaxel in MDA-MB-157 metaplastic breast cancer cells
Source: PLoS One. 2021 Aug 9;16(8):e0255738. doi: 10.1371/journal.pone.0255738 (PMC8351968; doi:10.1371/journal.pone.0255738)
Supplement: S5 Fig — (A) Hierarchical clustering and heat map of the genes associated with the over-represented biological process ontologies of cell cycle/cell division. Input data are the normalized expression values. The values in blue are upregulated and those in red are down-regulated. (B) The description of each gene in the clusters are available from the associated excel file. (PDF) [file pone.0255738.s005.pdf]

and Jenifer R. Prosperi

Supplementary Figure 2A. Cell cycle

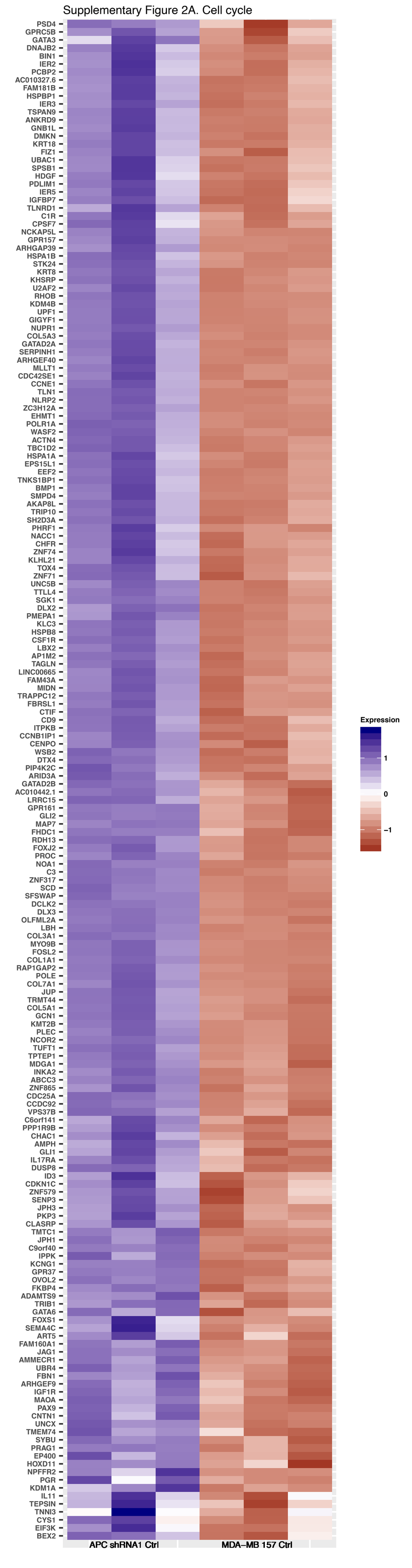

Supplemental Figure 2B. Up-regulated in the APC shRNA1 cells

ensembl_gene_id	hgnc	entrezgene_id	ids	baseMean	log2FoldChange	lfcSE	stat	pvalue	description
ENSG00000125637	PSD4	23550	190	45.62179794	1.660192691	0.303440068	5.47123753	4.47E-08	pleckstrin and Sec7 domain containing 4 [Source:HGNC Symbol;Acc:HGNC:19096]
ENSG00000167191	GPRC5B	51704	189	167.7575926	0.652971279	0.176337924	3.702954324	0.000213103	G protein-coupled receptor class C group 5 member B [Source:HGNC Symbol;Acc:HGNC:13308]
ENSG00000107485	GATA3	2625	188	11.8358029	2.908320678	0.656747381	4.428370424	9.49E-06	GATA binding protein 3 [Source:HGNC Symbol;Acc:HGNC:4172]
ENSG00000135924	DNAJB2	3300	187	1303.22337	1.032260733	0.153705497	6.715834857	1.87E-11	DnaJ heat shock protein family (Hsp40) member B2 [Source:HGNC Symbol;Acc:HGNC:5228]
ENSG00000136717	BIN1	274	186	1299.310413	1.059609053	0.162447847	6.522764506	6.9E-11	bridging integrator 1 [Source:HGNC Symbol;Acc:HGNC:1052]
ENSG00000160888	IER2	9592	185	910.6429647	0.961993417	0.137220009	7.010591421	2.37E-12	immediate early response 2 [Source:HGNC Symbol;Acc:HGNC:28871]
ENSG00000197111	PCBP2	5094	184	1777.330814	0.866911125	0.13415194	6.462158694	1.03E-10	poly(rC) binding protein 2 [Source:HGNC Symbol;Acc:HGNC:8648]
ENSG00000182103	FAM181B	220382	182	77.42158705	1.600935904	0.243823825	6.565953531	5.17E-11	family with sequence similarity 181 member B [Source:HGNC Symbol;Acc:HGNC:28512]
ENSG00000133265	HSPBP1	23640	181	4266.375207	1.607906019	0.240787799	6.677688937	2.43E-11	HSPA (Hsp70) binding protein 1 [Source:HGNC Symbol;Acc:HGNC:24989]
ENSG00000137331	IER3	8870	180	1456.42362	1.359737234	0.193857277	7.01411501	2.31E-12	immediate early response 3 [Source:HGNC Symbol;Acc:HGNC:5392]
ENSG00000011105	TSPAN9	10867	179	1856.714042	0.999488653	0.163670815	6.106700544	1.02E-09	tetraspanin 9 [Source:HGNC Symbol;Acc:HGNC:21640]
ENSG00000156381	ANKRD9	122416	178	797.6217251	1.183669393	0.179527272	6.593256735	4.3E-11	ankyrin repeat domain 9 [Source:HGNC Symbol;Acc:HGNC:20096]
ENSG00000185838	GNB1L	54584	177	391.030043	1.514355374	0.21705679	6.976770331	3.02E-12	G protein subunit beta 1 like [Source:HGNC Symbol;Acc:HGNC:4397]
ENSG00000161249	DMKN	93099	176	145.4444976	2.104584578	0.207812726	10.12731329	4.18E-24	dermokine [Source:HGNC Symbol;Acc:HGNC:25063]
ENSG00000111057	KRT18	3875	175	5691.2309	1.50734628	0.21142244	7.129547272	1.01E-12	keratin 18 [Source:HGNC Symbol;Acc:HGNC:6430]
ENSG00000179943	FIZ1	84922	174	512.6228392	1.096999165	0.167074301	6.565935978	5.17E-11	FLT3 interacting zinc finger 1 [Source:HGNC Symbol;Acc:HGNC:25917]
ENSG00000130560	UBAC1	10422	173	1089.035077	0.935638128	0.173082154	5.405745809	6.45E-08	UBA domain containing 1 [Source:HGNC Symbol;Acc:HGNC:30221]
ENSG00000171621	SPSB1	80176	172	178.8363239	0.994261804	0.185236302	5.367532127	7.98E-08	splA/ryanodine receptor domain and SOCS box containing 1 [Source:HGNC Symbol;Acc:HGNC:30628]
ENSG00000143321	HDGF	3068	171	6999.655947	1.033125855	0.191034668	5.408054293	6.37E-08	heparin binding growth factor [Source:HGNC Symbol;Acc:HGNC:4856]
ENSG00000107438	PDLIM1	9124	170	865.1782077	0.88881172	0.162496345	5.469733625	4.51E-08	PDZ and LIM domain 1 [Source:HGNC Symbol;Acc:HGNC:2067]
ENSG00000162783	IER5	51278	169	481.2622447	0.928163797	0.131730015	7.045955303	1.84E-12	immediate early response 5 [Source:HGNC Symbol;Acc:HGNC:5393]
ENSG00000163453	IGFBP7	3490	168	1050.9188	1.255526753	0.194878224	6.442622101	1.17E-10	insulin like growth factor binding protein 7 [Source:HGNC Symbol;Acc:HGNC:5476]
ENSG00000140406	TLNRD1	59274	167	336.6942218	0.858728046	0.180475572	4.75814004	1.95E-06	talin rod domain containing 1 [Source:HGNC Symbol;Acc:HGNC:13519]
ENSG00000159403	C1R	715	166	296.1074188	0.755463595	0.139619535	5.410873151	6.27E-08	complement C1r [Source:HGNC Symbol;Acc:HGNC:1246]
ENSG00000149532	CPSF7	79869	165	1176.154659	0.600368796	0.12727984	4.716919805	2.39E-06	cleavage and polyadenylation specific factor 7 [Source:HGNC Symbol;Acc:HGNC:30098]
ENSG00000167566	NCKAP5L	57701	164	785.5398031	1.099964327	0.201031824	5.471593027	4.46E-08	NCK associated protein 5 like [Source:HGNC Symbol;Acc:HGNC:29321]
ENSG00000180758	GPR157	80045	163	159.4055622	1.434396438	0.190778902	7.518632423	5.54E-14	G protein-coupled receptor 157 [Source:HGNC Symbol;Acc:HGNC:23687]
ENSG00000147799	ARHGAP39	80728	162	599.6921252	0.963451043	0.133862807	7.197301959	6.14E-13	Rho GTPase activating protein 39 [Source:HGNC Symbol;Acc:HGNC:29351]
ENSG00000204388	HSPA1B	3304	161	741.2424179	0.996211592	0.13624079	7.312138966	2.63E-13	heat shock protein family A (Hsp70) member 1B [Source:HGNC Symbol;Acc:HGNC:5233]
ENSG00000102572	STK24	8428	160	1064.780836	1.047225132	0.132584101	7.89857248	2.82E-15	serine/threonine kinase 24 [Source:HGNC Symbol;Acc:HGNC:11403]
ENSG00000170421	KRT8	3856	159	11184.29519	2.057155848	0.216173657	9.516218916	1.8E-21	keratin 8 [Source:HGNC Symbol;Acc:HGNC:6446]
ENSG00000088247	KHSRP	8570	158	6613.117588	1.010172278	0.141159739	7.156235112	8.29E-13	KH-type splicing regulatory protein [Source:HGNC Symbol;Acc:HGNC:6316]
ENSG00000063244	U2AF2	11338	157	2411.822057	1.089985736	0.158479517	6.877770442	6.08E-12	U2 small nuclear RNA auxiliary factor 2 [Source:HGNC Symbol;Acc:HGNC:23156]
ENSG00000143878	RHOB	388	156	1803.561265	1.353360115	0.161035654	8.404102315	4.31E-17	ras homolog family member B [Source:HGNC Symbol;Acc:HGNC:668]
ENSG00000127663	KDM4B	23030	155	1254.544064	1.139031722	0.151709371	7.507985253	6E-14	lysine demethylase 4B [Source:HGNC Symbol;Acc:HGNC:29136]
ENSG00000005007	UPF1	5976	154	2628.547066	1.106049532	0.129435158	8.545201718	1.28E-17	UPF1 RNA helicase and ATPase [Source:HGNC Symbol;Acc:HGNC:9962]
ENSG00000146830	GIGYF1	64599	153	602.5988307	0.941128597	0.172957495	5.44138662	5.29E-08	GRB10 interacting GYF protein 1 [Source:HGNC Symbol;Acc:HGNC:9126]
ENSG00000176046	NUPR1	26471	152	38.40806003	2.780059461	0.339889662	8.179299839	2.85E-16	nuclear protein 1, transcriptional regulator [Source:HGNC Symbol;Acc:HGNC:29990]
ENSG00000080573	COL5A3	50509	151	2864.394665	1.276319312	0.196288331	6.502267883	7.91E-11	collagen type V alpha 3 chain [Source:HGNC Symbol;Acc:HGNC:14864]
ENSG00000167491	GATAD2A	54815	150	1744.832396	0.739182419	0.11351198	6.511933072	7.42E-11	GATA zinc finger domain containing 2A [Source:HGNC Symbol;Acc:HGNC:29989]
ENSG00000149257	SERPINH1	871	149	687.1096701	1.549234094	0.153722393	10.07812891	6.9E-24	serpin family H member 1 [Source:HGNC Symbol;Acc:HGNC:1546]
ENSG00000165801	ARHGEF40	55701	148	1518.793642	0.966872332	0.16867194	5.732265449	9.91E-09	Rho guanine nucleotide exchange factor 40 [Source:HGNC Symbol;Acc:HGNC:25516]
ENSG00000130382	MLLT1	4298	147	1991.273017	0.94531464	0.141414864	6.684690811	2.31E-11	MLLT1 super elongation complex subunit [Source:HGNC Symbol;Acc:HGNC:7134]
ENSG00000197622	CDC42SE1	56882	146	438.2227431	0.741461416	0.12448065	5.956439144	2.58E-09	CDC42 small effector 1 [Source:HGNC Symbol;Acc:HGNC:17719]
ENSG00000105173	CCNE1	898	145	1767.45658	0.933998372	0.118399365	7.888542072	3.06E-15	cyclin E1 [Source:HGNC Symbol;Acc:HGNC:1589]
ENSG00000137076	TLN1	7094	144	7247.246479	1.019505907	0.181347538	5.621834832	1.89E-08	talin 1 [Source:HGNC Symbol;Acc:HGNC:11845]
ENSG00000022556	NLRP2	55655	143	1668.020727	1.115202414	0.137253706	8.125116952	4.47E-16	NLR family pyrin domain containing 2 [Source:HGNC Symbol;Acc:HGNC:22948]
ENSG00000163874	ZC3H12A	80149	142	116.595613	1.273498784	0.186660434	6.822542698	8.94E-12	zinc finger CCCH-type containing 12A [Source:HGNC Symbol;Acc:HGNC:26259]
ENSG00000181090	EHMT1	79813	141	515.3936471	0.901689932	0.123234551	7.316859819	2.54E-13	euchromatic histone lysine methyltransferase 1 [Source:HGNC Symbol;Acc:HGNC:24650]
ENSG00000068654	POLR1A	25885	140	1649.936837	0.818518279	0.125843102	6.504276105	7.81E-11	RNA polymerase I subunit A [Source:HGNC Symbol;Acc:HGNC:17264]
ENSG00000158195	WASF2	10163	139	2319.546168	0.804294252	0.147685148	5.446006338	5.15E-08	WASP family member 2 [Source:HGNC Symbol;Acc:HGNC:12733]
ENSG00000130402	ACTN4	81	138	15874.75795	1.082718285	0.14809047	7.311194887	2.65E-13	actinin alpha 4 [Source:HGNC Symbol;Acc:HGNC:166]
ENSG00000095383	TBC1D2	55357	137	543.688807	1.082627753	0.160022705	6.765463376	1.33E-11	TBC1 domain family member 2 [Source:HGNC Symbol;Acc:HGNC:18026]
ENSG00000204389	HSPA1A	3303	136	648.155884	1.051281021	0.155124304	6.77702328	1.23E-11	heat shock protein family A (Hsp70) member 1A [Source:HGNC Symbol;Acc:HGNC:5232]
ENSG00000127527	EPS15L1	58513	135	699.5602713	0.901388879	0.131439723	6.857811795	6.99E-12	epidermal growth factor receptor pathway substrate 15 like 1 [Source:HGNC Symbol;Acc:HGNC:24634]
ENSG00000167658	EEF2	1938	134	31866.47468	1.334498278	0.206356331	6.466960664	1E-10	eukaryotic translation elongation factor 2 [Source:HGNC Symbol;Acc:HGNC:3214]
ENSG00000149115	TNKS1BP1	85456	133	1531.399495	0.954432387	0.155365363	6.143147797	8.09E-10	tankyrase 1 binding protein 1 [Source:HGNC Symbol;Acc:HGNC:19081]
ENSG00000168487	BMP1	649	132	1311.931608	0.929367806	0.157083445	5.916395616	3.29E-09	bone morphogenetic protein 1 [Source:HGNC Symbol;Acc:HGNC:1067]
ENSG00000136699	SMPD4	55627	131	1370.301235	0.877320688	0.132146123	6.6390195	3.16E-11	sphingomyelin phosphodiesterase 4 [Source:HGNC Symbol;Acc:HGNC:32949]
ENSG00000011243	AKAP8L	26993	130	539.665454	0.931400348	0.113649391	8.195383548	2.5E-16	A-kinase anchoring protein 8 like [Source:HGNC Symbol;Acc:HGNC:29857]
ENSG00000125733	TRIP10	9322	129	1064.294161	1.171241461	0.150098524	7.803151064	6.04E-15	thyroid hormone receptor interactor 10 [Source:HGNC Symbol;Acc:HGNC:12304]
ENSG00000125731	SH2D3A	10045	128	318.2263808	1.29715983	0.209635139	6.187702294	6.1E-10	SH2 domain containing 3A [Source:HGNC Symbol;Acc:HGNC:16885]
ENSG00000070047	PHRF1	57661	127	1089.174035	0.7878242	0.143656781	5.484072483	4.16E-08	PHD and ring finger domains 1 [Source:HGNC Symbol;Acc:HGNC:24351]
ENSG00000160877	NACC1	112939	126	3220.685472	0.97775185	0.211869911	4.614868842	3.93E-06	nucleus accumbens associated 1 [Source:HGNC Symbol;Acc:HGNC:20967]
ENSG00000072609	CHFR	55743	125	707.7149467	0.8054214	0.164679028	4.890855923	1E-06	checkpoint with forkhead and ring finger domains [Source:HGNC Symbol;Acc:HGNC:20455]
ENSG00000185252	ZNF74	7625	124	358.1845307	1.004385131	0.15252455	6.585071919	4.55E-11	zinc finger protein 74 [Source:HGNC Symbol;Acc:HGNC:13144]
ENSG00000162413	KLHL21	9903	123	996.9344953	0.997100991	0.184918569	5.392108508	6.96E-08	kelch like family member 21 [Source:HGNC Symbol;Acc:HGNC:29041]
ENSG00000092203	TOX4	9878	122	1281.854095	0.853191109	0.139216999	6.128498064	8.87E-10	TOX high mobility group box family member 4 [Source:HGNC Symbol;Acc:HGNC:20161]
ENSG00000197951	ZNF71	58491	121	914.5294157	0.756456742	0.158490978	4.772869413	1.82E-06	zinc finger protein 71 [Source:HGNC Symbol;Acc:HGNC:13141]
ENSG00000107731	UNC5B	219699	120	80.36449343	1.306155109	0.238547343	5.475454447	4.36E-08	unc-5 netrin receptor B [Source:HGNC Symbol;Acc:HGNC:12568]
ENSG00000135912	TTLL4	9654	119	457.6656224	0.787611499	0.114953261	6.851580304	7.3E-12	tubulin tyrosine ligase like 4 [Source:HGNC Symbol;Acc:HGNC:28976]
ENSG00000118515	SGK1	6446	118	330.7357534	1.563926777	0.13846923	11.29439932	1.4E-29	serum/glucocorticoid regulated kinase 1 [Source:HGNC Symbol;Acc:HGNC:10810]
ENSG00000115844	DLX2	1746	117	264.1369877	1.201105157	0.120053186	10.00477536	1.45E-23	distal-less homeobox 2 [Source:HGNC Symbol;Acc:HGNC:2915]
ENSG00000124225	PMEPA1	56937	116	1145.495416	1.074854357	0.101826117	10.5557826	4.78E-26	prostate transmembrane protein, androgen induced 1 [Source:HGNC Symbol;Acc:HGNC:14107]
ENSG00000104892	KLC3	147700	115	144.7803119	2.171451177	0.297258609	7.304922753	2.77E-13	kinesin light chain 3 [Source:HGNC Symbol;Acc:HGNC:20717]
ENSG00000152137	HSPB8	26353	114	539.2141766	1.59380694	0.145399198	10.96159374	5.85E-28	heat shock protein family B (small) member 8 [Source:HGNC Symbol;Acc:HGNC:30171]
ENSG00000182578	CSF1R	1436	113	150.0862315	2.293112081	0.214798273	10.67565417	1.32E-26	colony stimulating factor 1 receptor [Source:HGNC Symbol;Acc:HGNC:2433]
ENSG00000179528	LBX2	85474	112	63.87962149	1.346400005	0.204460815	6.585124905	4.55E-11	ladybird homeobox 2 [Source:HGNC Symbol;Acc:HGNC:15525]
ENSG00000129354	AP1M2	10053	111	536.8694424	3.403717457	0.232371252	14.64775622	1.39E-48	adaptor related protein complex 1 subunit mu 2 [Source:HGNC Symbol;Acc:HGNC:558]
ENSG00000149591	TAGLN	6876	110	115.4047899	2.28427012	0.251174136	9.094368377	9.51E-20	transgelin [Source:HGNC Symbol;Acc:HGNC:11553]
ENSG00000232677	LINC00665	NA	109	747.9304703	0.917013183	0.142344248	6.44222155	1.18E-10	long intergenic non-protein coding RNA 665 [Source:HGNC Symbol;Acc:HGNC:44323]
ENSG00000185112	FAM43A	131583	108	310.8169162	1.189292041	0.164300505	7.238517242	4.54E-13	family with sequence similarity 43 member A [Source:HGNC Symbol;Acc:HGNC:26888]
ENSG00000167470	MIDN	90007	107	591.7008719	1.03868903	0.148315698	7.003230562	2.5E-12	midnolin [Source:HGNC Symbol;Acc:HGNC:16298]
ENSG00000171853	TRAPPC12	51112	106	782.9192803	0.691344937	0.10501994	6.582987366	4.61E-11	trafficking protein particle complex 12 [Source:HGNC Symbol;Acc:HGNC:24284]
ENSG00000112787	FBRSL1	57666	105	744.1731209	1.041128208	0.164876003	6.314613339	2.71E-10	fibrosin like 1 [Source:HGNC Symbol;Acc:HGNC:29308]
ENSG00000134030	CTIF	9811	104	360.9993854	1.157059414	0.178725328	6.473953231	9.55E-11	cap binding complex dependent translation initiation factor [Source:HGNC Symbol;Acc:HGNC:23925]
ENSG00000010278	CD9	928	103	2414.290141	0.982103466	0.127780312	7.68587469	1.52E-14	CD9 molecule [Source:HGNC Symbol;Acc:HGNC:1709]
ENSG00000143772	ITPKB	3707	102	551.5100192	1.224789968	0.169351667	7.232228576	4.75E-13	inositol-trisphosphate 3-kinase B [Source:HGNC Symbol;Acc:HGNC:6179]
ENSG00000100814	CCNB1IP1	57820	101	452.8707302	1.079276361	0.138249354	7.806737107	5.87E-15	cyclin B1 interacting protein 1 [Source:HGNC Symbol;Acc:HGNC:19437]
ENSG00000138092	CENPO	79172	100	407.0570619	0.834453331	0.127115437	6.564531817	5.22E-11	centromere protein O [Source:HGNC Symbol;Acc:HGNC:28152]
ENSG00000176871	WSB2	55884	99	845.5937797	0.863633617	0.120679588	7.156418347	8.28E-13	WD repeat and SOCS box containing 2 [Source:HGNC Symbol;Acc:HGNC:19222]
ENSG00000110042	DTX4	23220	98	254.3651069	1.20896722	0.152613042	7.921781804	2.34E-15	deltex E3 ubiquitin ligase 4 [Source:HGNC Symbol;Acc:HGNC:29151]
ENSG00000166908	PIP4K2C	79837	97	604.9381354	0.84945359	0.118982834	7.139295314	9.38E-13	phosphatidylinositol-5-phosphate 4-kinase type 2 gamma [Source:HGNC Symbol;Acc:HGNC:23786]
ENSG00000116017	ARID3A	1820	96	194.4276165	0.692102123	0.12919195	5.357161365	8.45E-08	AT-rich interaction domain 3A [Source:HGNC Symbol;Acc:HGNC:3031]
ENSG00000143614	GATAD2B	57459	95	306.3068155	1.077411234	0.146421742	7.35827357	1.86E-13	GATA zinc finger domain containing 2B [Source:HGNC Symbol;Acc:HGNC:30778]
ENSG00000172061	LRRC15	131578	93	22.24335376	1.262599204	0.410947172	3.072412443	0.002123361	leucine rich repeat containing 15 [Source:HGNC Symbol;Acc:HGNC:20818]
ENSG00000143147	GPR161	23432	92	509.1292255	0.693606906	0.106043302	6.540789391	6.12E-11	G protein-coupled receptor 161 [Source:HGNC Symbol;Acc:HGNC:23694]
ENSG00000074047	GLI2	2736	91	260.275435	1.057268164	0.135561967	7.799150364	6.23E-15	GLI family zinc finger 2 [Source:HGNC Symbol;Acc:HGNC:4318]
ENSG00000135525	MAP7	9053	90	64.93662682	1.241539863	0.243111705	5.106869966	3.28E-07	microtubule associated protein 7 [Source:HGNC Symbol;Acc:HGNC:6869]
ENSG00000137460	FHDC1	85462	89	148.2468776	0.850269764	0.138376916	6.144592522	8.02E-10	FH2 domain containing 1 [Source:HGNC Symbol;Acc:HGNC:29363]
ENSG00000160439	RDH13	112724	88	78.81327864	1.491986578	0.229832268	6.491632304	8.49E-11	retinol dehydrogenase 13 [Source:HGNC Symbol;Acc:HGNC:19978]
ENSG00000065970	FOXJ2	55810	87	433.9356574	0.953713376	0.11020858	8.6537126	4.99E-18	forkhead box J2 [Source:HGNC Symbol;Acc:HGNC:24818]
ENSG00000115718	PROC	5624	86	85.41013936	1.751627585	0.211715288	8.273505428	1.3E-16	protein C, inactivator of coagulation factors Va and VIIIa [Source:HGNC Symbol;Acc:HGNC:9451]
ENSG00000084092	NOA1	84273	85	208.3261986	0.749601456	0.110880037	6.760472629	1.38E-11	nitric oxide associated 1 [Source:HGNC Symbol;Acc:HGNC:28473]
ENSG00000125730	C3	718	84	138.4209353	2.720455203	0.205586638	13.23264597	5.69E-40	complement C3 [Source:HGNC Symbol;Acc:HGNC:1318]
ENSG00000130803	ZNF317	57693	83	723.5033937	0.78072636	0.142866725	5.464717955	4.64E-08	zinc finger protein 317 [Source:HGNC Symbol;Acc:HGNC:13507]
ENSG00000099194	SCD	6319	82	1062.046411	1.145640948	0.12641425	9.062593384	1.27E-19	stearoyl-CoA desaturase [Source:HGNC Symbol;Acc:HGNC:10571]
ENSG00000061936	SFSWAP	6433	81	483.4566095	0.692298114	0.100595202	6.882019232	5.9E-12	splicing factor SWAP [Source:HGNC Symbol;Acc:HGNC:10790]
ENSG00000170390	DCLK2	166614	80	319.2411877	1.098210437	0.115031341	9.547054098	1.33E-21	doublecortin like kinase 2 [Source:HGNC Symbol;Acc:HGNC:19002]
ENSG00000064195	DLX3	1747	79	59.42044107	4.988673607	0.38213048	13.05489581	5.96E-39	distal-less homeobox 3 [Source:HGNC Symbol;Acc:HGNC:2916]
ENSG00000185585	OLFML2A	169611	78	504.344028	1.204466845	0.155524375	7.744553522	9.59E-15	olfactomedin like 2A [Source:HGNC Symbol;Acc:HGNC:27270]
ENSG00000213626	LBH	81606	77	393.9340595	1.933618283	0.158307491	12.21431958	2.61E-34	LBH regulator of WNT signaling pathway [Source:HGNC Symbol;Acc:HGNC:29532]
ENSG00000168542	COL3A1	1281	76	4242.726117	1.217824864	0.13612776	8.94619044	3.68E-19	collagen type III alpha 1 chain [Source:HGNC Symbol;Acc:HGNC:2201]
ENSG00000099331	MYO9B	4650	75	1943.136863	1.007672637	0.150038956	6.716073361	1.87E-11	myosin IXB [Source:HGNC Symbol;Acc:HGNC:7609]
ENSG00000075426	FOSL2	2355	74	1677.466794	0.794340015	0.114332568	6.947626843	3.71E-12	FOS like 2, AP-1 transcription factor subunit [Source:HGNC Symbol;Acc:HGNC:3798]
ENSG00000108821	COL1A1	1277	73	13428.53979	2.278951309	0.151990583	14.99402963	8.03E-51	collagen type I alpha 1 chain [Source:HGNC Symbol;Acc:HGNC:2197]
ENSG00000132359	RAP1GAP2	23108	72	81.4164133	1.410042851	0.185357024	7.607172485	2.8E-14	RAP1 GTPase activating protein 2 [Source:HGNC Symbol;Acc:HGNC:29176]
ENSG00000177084	POLE	5426	71	1541.387807	0.741268572	0.122353763	6.058404372	1.37E-09	DNA polymerase epsilon, catalytic subunit [Source:HGNC Symbol;Acc:HGNC:9177]
ENSG00000114270	COL7A1	1294	70	299.3661705	1.480704195	0.171031315	8.657503419	4.82E-18	collagen type VII alpha 1 chain [Source:HGNC Symbol;Acc:HGNC:2214]
ENSG00000173801	JUP	3728	69	1187.589784	1.721318691	0.204134061	8.432295347	3.39E-17	junction plakoglobin [Source:HGNC Symbol;Acc:HGNC:6207]
ENSG00000155275	TRMT44	152992	68	136.0203497	0.802420026	0.147850411	5.427242446	5.72E-08	tRNA methyltransferase 44 homolog [Source:HGNC Symbol;Acc:HGNC:26653]
ENSG00000130635	COL5A1	1289	67	6681.072462	0.891815812	0.163942081	5.439822441	5.33E-08	collagen type V alpha 1 chain [Source:HGNC Symbol;Acc:HGNC:2209]
ENSG00000089154	GCN1	10985	66	3660.723974	0.961292086	0.166200578	5.783927454	7.3E-09	GCN1 activator of EIF2AK4 [Source:HGNC Symbol;Acc:HGNC:4199]
ENSG00000272333	KMT2B	9757	65	2019.499207	0.965050699	0.170905841	5.646680614	1.64E-08	lysine methyltransferase 2B [Source:HGNC Symbol;Acc:HGNC:15840]
ENSG00000178209	PLEC	5339	64	17189.12856	1.216142565	0.178793634	6.801934388	1.03E-11	plectin [Source:HGNC Symbol;Acc:HGNC:9069]
ENSG00000196498	NCOR2	9612	63	2989.320989	1.22428167	0.141464278	8.654352114	4.96E-18	nuclear receptor corepressor 2 [Source:HGNC Symbol;Acc:HGNC:7673]
ENSG00000143367	TUFT1	7286	62	440.9239551	1.109804942	0.139412166	7.960603255	1.71E-15	tuftelin 1 [Source:HGNC Symbol;Acc:HGNC:12422]
ENSG00000100181	TPTEP1	NA	61	269.1399771	1.246449786	0.1956066	6.372227646	1.86E-10	TPTE pseudogene 1 [Source:HGNC Symbol;Acc:HGNC:43648]
ENSG00000112139	MDGA1	266727	60	309.5652374	0.788042447	0.18067075	4.361759976	1.29E-05	MAM domain containing glycosylphosphatidylinositol anchor 1 [Source:HGNC Symbol;Acc:HGNC:19267]
ENSG00000197852	INKA2	55924	59	103.3970802	1.436885167	0.173154252	8.298295622	1.06E-16	inka box actin regulator 2 [Source:HGNC Symbol;Acc:HGNC:28045]
ENSG00000108846	ABCC3	8714	58	229.4728387	1.070544785	0.144530846	7.407033276	1.29E-13	ATP binding cassette subfamily C member 3 [Source:HGNC Symbol;Acc:HGNC:54]
ENSG00000261221	ZNF865	100507290	57	441.9159814	0.810628111	0.147735136	5.487036674	4.09E-08	zinc finger protein 865 [Source:HGNC Symbol;Acc:HGNC:38705]
ENSG00000164045	CDC25A	993	56	278.9716048	0.882186013	0.160804267	5.486085852	4.11E-08	cell division cycle 25A [Source:HGNC Symbol;Acc:HGNC:1725]
ENSG00000119242	CCDC92	80212	55	340.8477979	0.952396665	0.117775774	8.086524369	6.14E-16	coiled-coil domain containing 92 [Source:HGNC Symbol;Acc:HGNC:29563]
ENSG00000139722	VPS37B	79720	54	151.1529658	0.966868276	0.220759345	4.379738835	1.19E-05	VPS37B subunit of ESCRT-I [Source:HGNC Symbol;Acc:HGNC:25754]
ENSG00000197261	C6orf141	135398	53	29.62628739	1.402333461	0.319447676	4.389869037	1.13E-05	chromosome 6 open reading frame 141 [Source:HGNC Symbol;Acc:HGNC:21351]
ENSG00000108819	PPP1R9B	84687	52	370.0997182	1.093376704	0.12704216	8.606408352	7.54E-18	protein phosphatase 1 regulatory subunit 9B [Source:HGNC Symbol;Acc:HGNC:9298]
ENSG00000128965	CHAC1	79094	51	142.2079899	1.590197681	0.206773824	7.690517345	1.47E-14	ChaC glutathione specific gamma-glutamylcyclotransferase 1 [Source:HGNC Symbol;Acc:HGNC:28680]
ENSG00000078053	AMPH	273	50	6.69595942	2.181220151	0.539070486	4.046261496	5.2E-05	amphiphysin [Source:HGNC Symbol;Acc:HGNC:471]
ENSG00000111087	GLI1	2735	49	7.078567159	1.989019126	0.629371331	3.160326868	0.001575922	GLI family zinc finger 1 [Source:HGNC Symbol;Acc:HGNC:4317]
ENSG00000177663	IL17RA	23765	48	366.6536655	0.751653707	0.116527133	6.450460842	1.12E-10	interleukin 17 receptor A [Source:HGNC Symbol;Acc:HGNC:5985]
ENSG00000184545	DUSP8	1850	47	222.6032711	1.413796358	0.191044136	7.400365111	1.36E-13	dual specificity phosphatase 8 [Source:HGNC Symbol;Acc:HGNC:3074]
ENSG00000117318	ID3	3399	46	2020.029432	1.293386902	0.207746958	6.225780226	4.79E-10	inhibitor of DNA binding 3, HLH protein [Source:HGNC Symbol;Acc:HGNC:5362]
ENSG00000129757	CDKN1C	1028	45	234.1320258	1.318604603	0.209773985	6.28583474	3.26E-10	cyclin dependent kinase inhibitor 1C [Source:HGNC Symbol;Acc:HGNC:1786]
ENSG00000218891	ZNF579	163033	44	296.6102983	1.063731382	0.219839368	4.838675581	1.31E-06	zinc finger protein 579 [Source:HGNC Symbol;Acc:HGNC:26646]
ENSG00000161956	SENP3	26168	43	306.344743	0.942181319	0.206356444	4.565795473	4.98E-06	SUMO specific peptidase 3 [Source:HGNC Symbol;Acc:HGNC:17862]
ENSG00000154118	JPH3	57338	42	218.0327488	1.238019506	0.152967829	8.093332514	5.81E-16	junctophilin 3 [Source:HGNC Symbol;Acc:HGNC:14203]
ENSG00000184363	PKP3	11187	41	150.9534706	1.293751562	0.233832377	5.532816199	3.15E-08	plakophilin 3 [Source:HGNC Symbol;Acc:HGNC:9025]
ENSG00000104859	CLASRP	11129	40	338.1871868	0.819740229	0.151158494	5.423051042	5.86E-08	CLK4 associating serine/arginine rich protein [Source:HGNC Symbol;Acc:HGNC:17731]
ENSG00000133687	TMTC1	83857	39	40.69140566	2.653723656	0.28983026	9.15613042	5.38E-20	transmembrane O-mannosyltransferase targeting cadherins 1 [Source:HGNC Symbol;Acc:HGNC:24099]
ENSG00000104369	JPH1	56704	38	16.53881553	3.212844046	0.375316396	8.560361554	1.13E-17	junctophilin 1 [Source:HGNC Symbol;Acc:HGNC:14201]
ENSG00000135045	C9orf40	55071	37	213.3245278	0.826044072	0.130252163	6.341883701	2.27E-10	chromosome 9 open reading frame 40 [Source:HGNC Symbol;Acc:HGNC:23433]
ENSG00000127080	IPPK	64768	36	132.4965045	0.966092741	0.148157037	6.520734773	7E-11	inositol-pentakisphosphate 2-kinase [Source:HGNC Symbol;Acc:HGNC:14645]
ENSG00000026559	KCNG1	3755	35	112.3700685	1.282755789	0.192102203	6.677465259	2.43E-11	potassium voltage-gated channel modifier subfamily G member 1 [Source:HGNC Symbol;Acc:HGNC:6248]
ENSG00000170775	GPR37	2861	34	76.09740216	2.47113435	0.1970425	12.54112359	4.45E-36	G protein-coupled receptor 37 [Source:HGNC Symbol;Acc:HGNC:4494]
ENSG00000125850	OVOL2	58495	33	9.091588028	2.585898117	0.575754998	4.49131684	7.08E-06	ovo like zinc finger 2 [Source:HGNC Symbol;Acc:HGNC:15804]
ENSG00000004478	FKBP4	2288	32	103.3631834	1.885065625	0.250077675	7.537920483	4.78E-14	FKBP prolyl isomerase 4 [Source:HGNC Symbol;Acc:HGNC:3720]
ENSG00000163638	ADAMTS9	56999	31	3.412174648	5.233127714	1.176507852	4.44801767	8.67E-06	ADAM metallopeptidase with thrombospondin type 1 motif 9 [Source:HGNC Symbol;Acc:HGNC:13202]
ENSG00000173334	TRIB1	10221	30	178.6359857	1.045881476	0.123061046	8.498883362	1.91E-17	tribbles pseudokinase 1 [Source:HGNC Symbol;Acc:HGNC:16891]
ENSG00000141448	GATA6	2627	29	25.36469883	1.764845179	0.324726199	5.434871541	5.48E-08	GATA binding protein 6 [Source:HGNC Symbol;Acc:HGNC:4174]
ENSG00000179772	FOXS1	2307	28	3.000706109	5.131510673	1.372356683	3.73919604	0.00018461	forkhead box S1 [Source:HGNC Symbol;Acc:HGNC:3735]
ENSG00000168758	SEMA4C	54910	27	301.4486508	0.638261605	0.158473935	4.027549417	5.64E-05	semaphorin 4C [Source:HGNC Symbol;Acc:HGNC:10731]
ENSG00000167311	ART5	116969	26	188.0959515	0.985842425	0.22048902	4.471163342	7.78E-06	ADP-ribosyltransferase 5 [Source:HGNC Symbol;Acc:HGNC:24049]
ENSG00000164142	FAM160A1	729830	25	57.82135282	1.49260491	0.212905464	7.010646346	2.37E-12	family with sequence similarity 160 member A1 [Source:HGNC Symbol;Acc:HGNC:34237]
ENSG00000101384	JAG1	182	24	458.7934686	1.130904856	0.138761258	8.150004331	3.64E-16	jagged canonical Notch ligand 1 [Source:HGNC Symbol;Acc:HGNC:6188]
ENSG00000101935	AMMECR1	9949	23	73.17997069	1.422922915	0.244547175	5.818602943	5.93E-09	AMMECR nuclear protein 1 [Source:HGNC Symbol;Acc:HGNC:467]
ENSG00000127481	UBR4	23352	22	3547.727977	0.985001824	0.187840628	5.243816697	1.57E-07	ubiquitin protein ligase E3 component n-recognin 4 [Source:HGNC Symbol;Acc:HGNC:30313]
ENSG00000166147	FBN1	2200	21	194.4033605	1.346182529	0.211769459	6.356830379	2.06E-10	fibrillin 1 [Source:HGNC Symbol;Acc:HGNC:3603]
ENSG00000131089	ARHGEF9	23229	20	54.45171444	0.848474027	0.198047564	4.284193206	1.83E-05	Cdc42 guanine nucleotide exchange factor 9 [Source:HGNC Symbol;Acc:HGNC:14561]
ENSG00000140443	IGF1R	3480	19	167.0156969	0.902804039	0.170046709	5.309153261	1.1E-07	insulin like growth factor 1 receptor [Source:HGNC Symbol;Acc:HGNC:5465]
ENSG00000189221	MAOA	4128	18	7.734354412	2.114090704	0.511408088	4.133862473	3.57E-05	monoamine oxidase A [Source:HGNC Symbol;Acc:HGNC:6833]
ENSG00000198807	PAX9	5083	17	14.53664829	1.770484949	0.414445093	4.27194091	1.94E-05	paired box 9 [Source:HGNC Symbol;Acc:HGNC:8623]
ENSG00000018236	CNTN1	1272	16	2.102580254	5.017383551	1.405960133	3.568652791	0.000358822	contactin 1 [Source:HGNC Symbol;Acc:HGNC:2171]
ENSG00000164853	UNCX	340260	15	19.51546183	1.756483252	0.368007155	4.772959513	1.82E-06	UNC homeobox [Source:HGNC Symbol;Acc:HGNC:33194]
ENSG00000164841	TMEM74	157753	14	4.85552181	3.013458384	0.822137796	3.665393317	0.000246959	transmembrane protein 74 [Source:HGNC Symbol;Acc:HGNC:26409]
ENSG00000147642	SYBU	55638	13	103.8509184	1.419825494	0.194878197	7.285707259	3.2E-13	syntabulin [Source:HGNC Symbol;Acc:HGNC:26011]
ENSG00000275342	PRAG1	157285	12	300.7896079	0.891127863	0.142420997	6.256997779	3.92E-10	PEAK1 related, kinase-activating pseudokinase 1 [Source:HGNC Symbol;Acc:HGNC:25438]
ENSG00000183495	EP400	57634	11	635.4565309	0.845401208	0.168795553	5.008432951	5.49E-07	E1A binding protein p400 [Source:HGNC Symbol;Acc:HGNC:11958]
ENSG00000128713	HOXD11	3237	10	6.161741011	1.917847213	0.805140899	2.382001976	0.017218805	homeobox D11 [Source:HGNC Symbol;Acc:HGNC:5134]
ENSG00000056291	NPFFR2	10886	9	29.09294225	0.899811384	0.244428222	3.681290877	0.000232056	neuropeptide FF receptor 2 [Source:HGNC Symbol;Acc:HGNC:4525]
ENSG00000082175	PGR	5241	8	37.11208819	1.193295139	0.311344267	3.832719164	0.000126735	progesterone receptor [Source:HGNC Symbol;Acc:HGNC:8910]
ENSG00000004487	KDM1A	23028	7	9.711064788	2.351736019	0.603116603	3.899305717	9.65E-05	lysine demethylase 1A [Source:HGNC Symbol;Acc:HGNC:29079]
ENSG00000095752	IL11	3589	6	4417.107715	0.845635048	0.199191197	4.245343485	2.18E-05	interleukin 11 [Source:HGNC Symbol;Acc:HGNC:5966]
ENSG00000167302	TEPSIN	146705	5	160.0140229	0.72492128	0.198945938	3.643810414	0.000268631	TEPSIN adaptor related protein complex 4 accessory protein [Source:HGNC Symbol;Acc:HGNC:26458]
ENSG00000129991	TNNI3	7137	4	10.14967577	1.66230987	0.84045026	1.977880129	0.047942231	troponin I3, cardiac type [Source:HGNC Symbol;Acc:HGNC:11947]
ENSG00000205795	CYS1	192668	3	243.1573982	0.666973049	0.188509173	3.538146385	0.000402947	cystin 1 [Source:HGNC Symbol;Acc:HGNC:18525]
ENSG00000178982	EIF3K	27335	2	7248.019048	0.707772976	0.165627218	4.273289041	1.93E-05	eukaryotic translation initiation factor 3 subunit K [Source:HGNC Symbol;Acc:HGNC:24656]
ENSG00000133134	BEX2	84707	1	122.2473932	0.616108683	0.159963273	3.85156338	0.000117366	brain expressed X-linked 2 [Source:HGNC Symbol;Acc:HGNC:30933]

1

2
