# Supplementary material for: Adenomatous Polyposis Coli loss controls cell cycle regulators and response to paclitaxel in MDA-MB-157 metaplastic breast cancer cells
Source: PLoS One. 2021 Aug 9;16(8):e0255738. doi: 10.1371/journal.pone.0255738 (PMC8351968; doi:10.1371/journal.pone.0255738)

Full western blots from Figure 2.

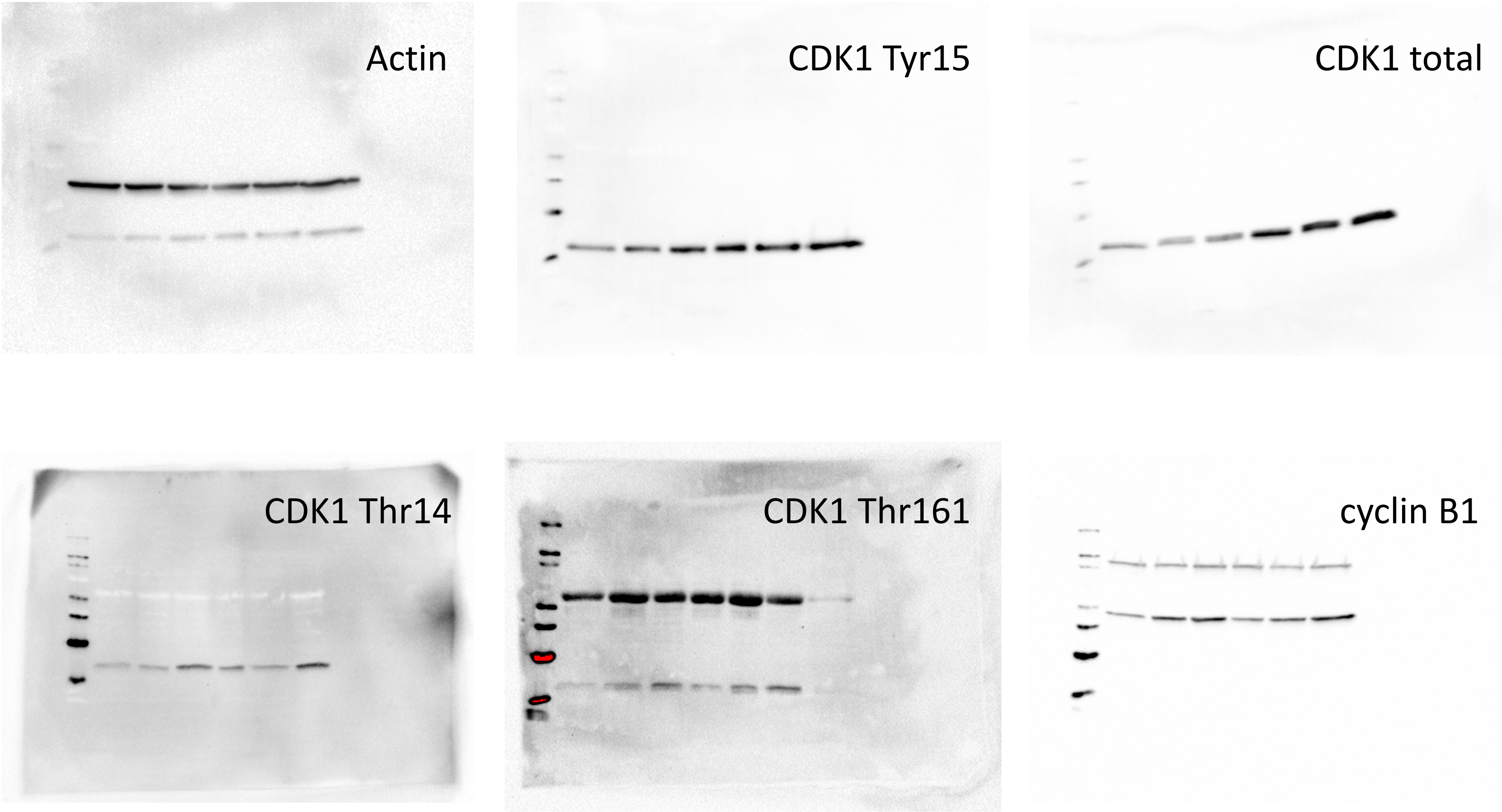

Full western blots from Figure 3.

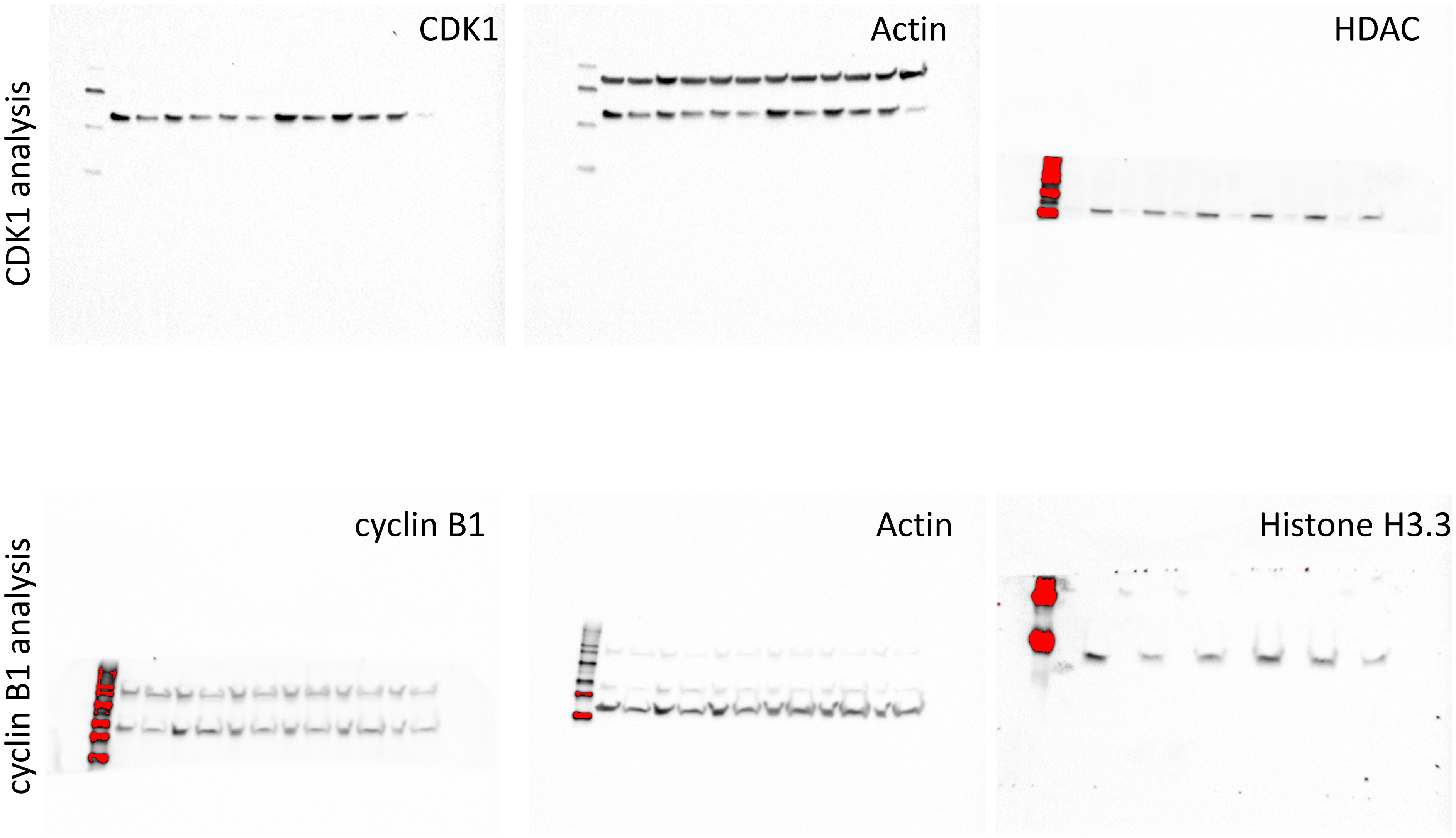

Full western blots from Figure 4.

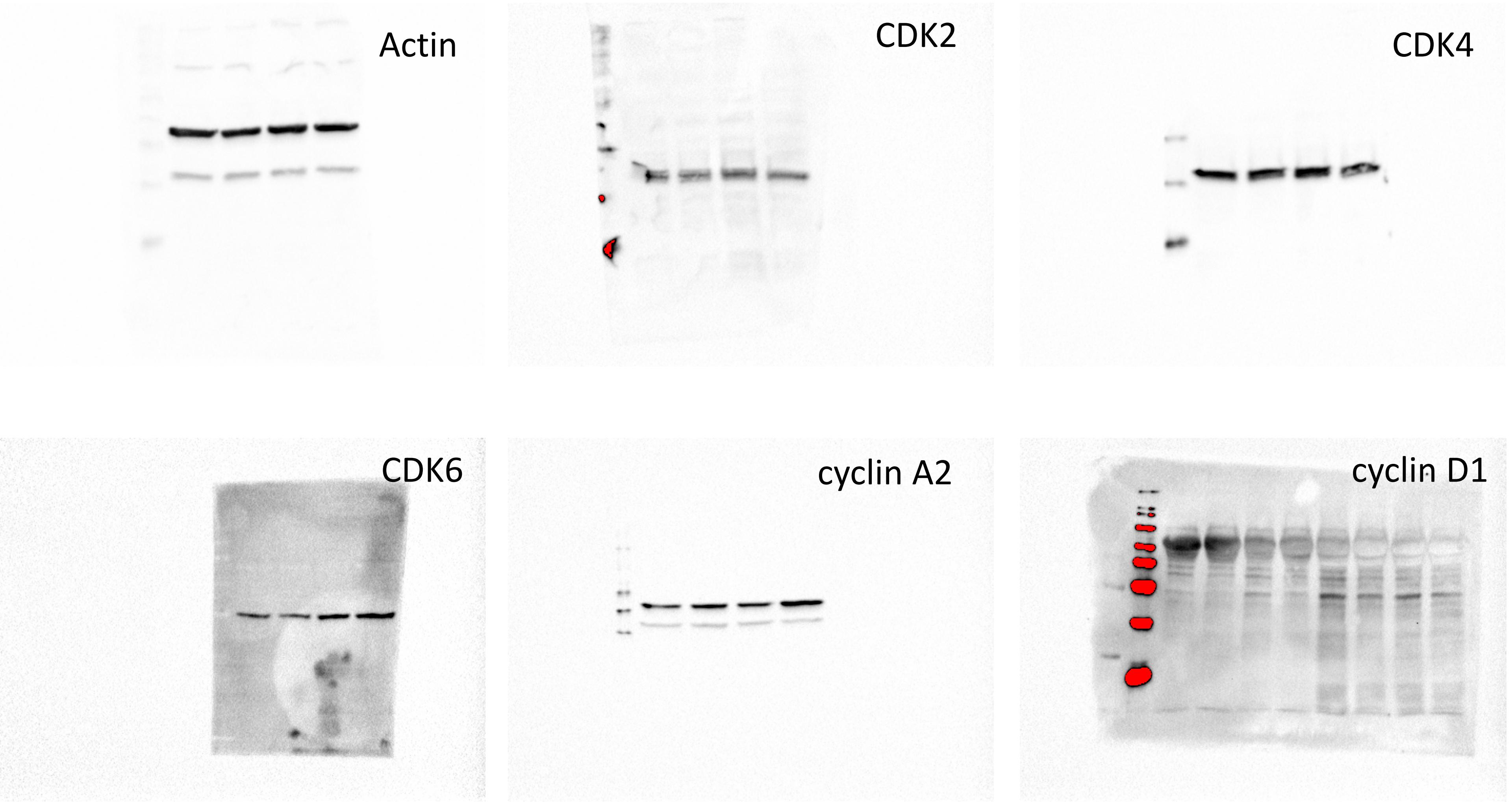

Full western blots from Figure 4 continued.

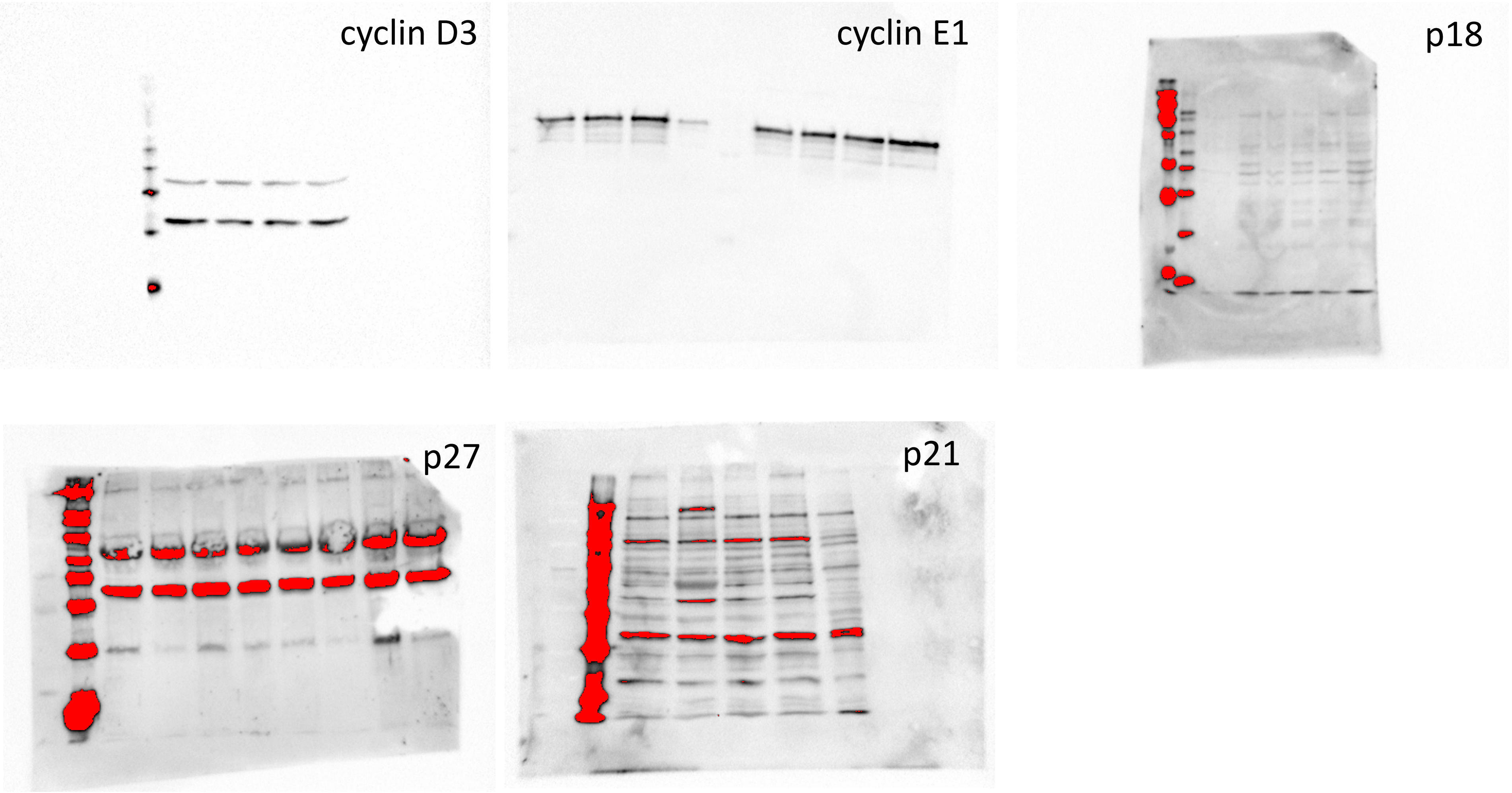

Full western blots from Figure 5.

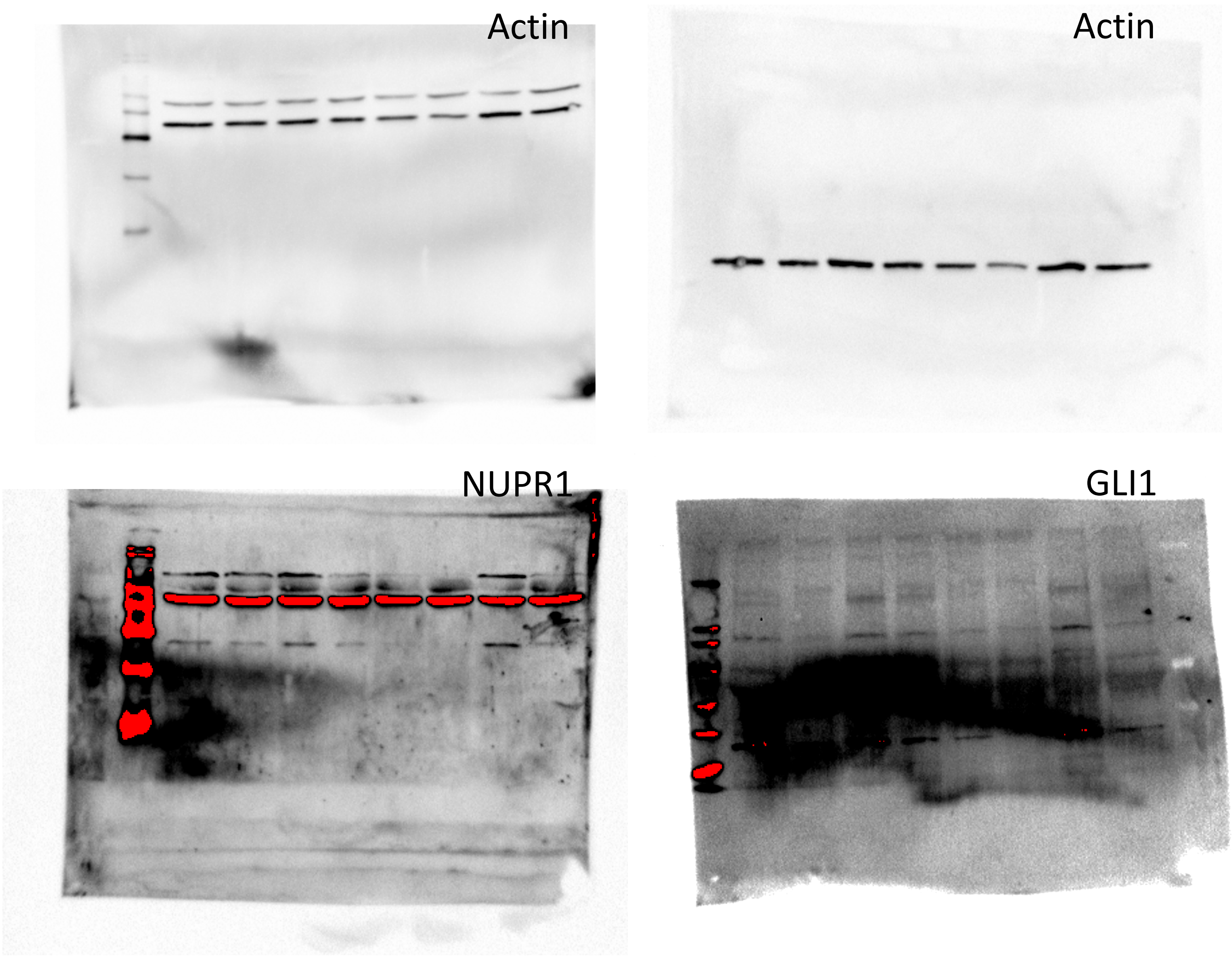

Supplement: S1 Raw images — (PDF) [file pone.0255738.s006.pdf]
