# Supplementary material for: Adenomatous Polyposis Coli loss controls cell cycle regulators and response to paclitaxel in MDA-MB-157 metaplastic breast cancer cells
Source: PLoS One. 2021 Aug 9;16(8):e0255738. doi: 10.1371/journal.pone.0255738 (PMC8351968; doi:10.1371/journal.pone.0255738)
Supplement: Attachment — Submitted filename: Response to Reviewers.docx [file pone.0255738.s007.docx]

**Editorial Review**

1. Please ensure that your manuscript meets PLOS ONE's style requirements, including those for file naming. The PLOS ONE style templates can be found at

<https://journals.plos.org/plosone/s/file?id=wjVg/PLOSOne_formatting_sample_main_body.pdf> and

[https://journals.plos.org/plosone/s/file?id=ba62/PLOSOne_formatting_sample_title_authors_affiliations.pdf](about:blank)

Changes have been made to ensure that the manuscript follows the PLOS ONE style requirements.

2. We note that you have included the phrase “data not shown” in your manuscript. Unfortunately, this does not meet our data sharing requirements. PLOS does not permit references to inaccessible data. We require that authors provide all relevant data within the paper, Supporting Information files, or in an acceptable, public repository. Please add a citation to support this phrase or upload the data that corresponds with these findings to a stable repository (such as Figshare or Dryad) and provide and URLs, DOIs, or accession numbers that may be used to access these data. Or, if the data are not a core part of the research being presented in your study, we ask that you remove the phrase that refers to these data. These data were not a core part of the research in the current study and have therefore been removed.

3. PLOS ONE now requires that authors provide the original uncropped and unadjusted images underlying all blot or gel results reported in a submission’s figures or Supporting Information files. This policy and the journal’s other requirements for blot/gel reporting and figure preparation are described in detail at <https://journals.plos.org/plosone/s/figures#loc-blot-and-gel-reporting-requirements> and <https://journals.plos.org/plosone/s/figures#loc-preparing-figures-from-image-files>. When you submit your revised manuscript, please ensure that your figures adhere fully to these guidelines and provide the original underlying images for all blot or gel data reported in your submission. See the following link for instructions on providing the original image data: <https://journals.plos.org/plosone/s/figures#loc-original-images-for-blots-and-gels>.

In your cover letter, please note whether your blot/gel image data are in Supporting Information or posted at a public data repository, provide the repository URL if relevant, and provide specific details as to which raw blot/gel images, if any, are not available. Email us at [plosone@plos.org](mailto:plosone@plos.org) if you have any questions.

We have included a PDF document of the raw gel images in the supplementary information.

**Reviewer 1**

1. Most of the data are reported in this paper on the same cell line as breast:
APC selectively mediates response to chemotherapeutic agents in breast cancer.
VanKlompenberg MK, Bedalov CO, Soto KF, Prosperi JR.BMC Cancer. 2016 Nov 28;16(1):921. doi: 10.1186/s12885-016-2950-5. We appreciate this reviewers concern, but the data presented here are novel and have not been previously published. We have built off the initial finding that the MDA-MB-157 cells with shRNA-mediated knockdown of APC results in paclitaxel resistance, which was in the VanKlompenberg et al, 2015 paper. In the current manuscript, however, we have expanded to understand the molecular underpinnings of the taxane resistance found in these cells.

2. I think that the paper is not suitable for the publication in the present form, most of the experiments are not convincing and regarding the statistical analysis I have several doubts about the tests used p value with **** does not exist. All of our statistical analysis has been double and sometimes triple checked by separate authors of the manuscript.

**Reviewer 2**

1. The study has considered single TNBC cell line MD-MB-157 to study the loss of APC in the resistance to Paclitaxel. Experiments should be performed in at least two or more APC modified TNBC cell lines.

While we appreciate the comment about performing experiments in multiple cell lines, to the best of our knowledge, this is not a requirement of PLOS ONE. We are currently working on generating CRISPR-mediated APC knockout cell lines using multiple TNBC cell lines, including CRISPR-mediated knockout in the MDA-MB-157 line. It is our intention that we will use these cells as a panel in future studies with combination therapy.

Furthermore, cell proliferation assay and annexin staining should be shown in the manuscript in both parental and APC modified cell line with and without Paclitaxel treatment.

Cell proliferation has been shown in the VanKlompenberg et al, 2015 paper (Fig 5C). In line with the cleaved caspase 3 we showed in VanKlompenberg et al 2015, we have also included annexin/PI analysis of apoptosis here as Supplemental Figure 2, and the methods are included (lines 127-132).

2. The blot for CDK1 Thr161, an important marker of CDK1 activation is not visible at all. Also, please indicate the molecular size of the bands. We have provided a better representative image clearly showing the CDK1 Thr161 expression in Fig 2A. In addition, we have now included the molecular size of each protein in Table 1, along with the antibody information.

3. Relative expression changes of CDK6 and p27 calculated in Figure 4 do not match visibly with that of the representative blot. Expression of p27 in the APC shRNA CTL lysates appear decreased compared to parental untreated CTL lysates. The authors should include an explanation for the decrease levels of CDK6 and p27 levels observed on Paclitaxel treatment in the APC modified lines. We apologize for any perceived inconsistencies and have confirmed that all images are the best representative images. To address the specific comments here: There is no change in the expression of p27 between the APC shRNA CTL and parental CTL treated cells. The CDK6 levels are unchanged with PTX treatment in either cell line. We have included a brief comment on the decreased expression of p27 by PTX in the discussion (lines 417-423).

4. Again, blot of Paclitaxel treated parental lines (GLI1 and NUPR1) from Figure 5C does not match with the relative expression levels quantified. While we chose representative blots for the initial submission, we have provided different representative blots that more closely match the quantification.

5. The author should provide an explanation for no significant changes seen in the GLI1 and NUPR1 levels of Paclitaxel treated parental lines? The RNA-sequencing data analysis that we are showing is depicting changes between the parental and APC shRNA cell lines. We haven’t shown any data to suggest that PTX impacts the expression of GLI1 and NUPR1. In fact, in speaking to the point below, we hypothesize (for future studies) that alterations of the expression of GLI1 and NUPR1 may change the response to PTX. In short, we have shown that these are downstream of APC loss, but not PTX-mediated expression changes.

6. Knockdown or over expression studies of cell cycle regulators identified via RNA seq need to be majorly performed in APC modified or parental lines proving loss of APC and the likely mechanism of resistance in response to Paclitaxel. While we appreciate this comment, these experiments are underway in the laboratory and will serve as the basis of an additional manuscript.

**Reviewer 3**

1.Major concerns:

1.1 All experiments in this work are performed on MDA-MB-157 and its clonal derivative, APC shRNA cells. This raises a concern if the reported effects of Apc loss are unique to this cell line. Is MDA-MB-157 a representative cell line of all TNBCs? If yes, then sufficient proof by providing references from earlier works should be provided. If no, a few other TNBC cell lines need to be included. Else, the title is not justified. One suggestion to take care of this concern, is to include at least two other TNBC cell lines and a “normal” breast cell line such as MCF-10A and compare the expression profiles of the reported genes by using WB/qPCR following siRNA APC knockdown. Some of these concerns are addressed above in the response to Reviewer 2 (point 1). In addition, the MDA-MB-157 cells are of the metaplastic subgroup of TNBC, and were specifically chosen because of this subgrouping. This has been addressed in the VanKlompenberg et al (2015) paper, and we have now included an explanation in the Introduction (lines 73-78).

1.2 The authors make an important observation (supplementary figure S2 and line 301) that Apc loss does not activate the Wnt beta-catenin dependent pathway in MDA-MB-157 cells and MMTV-PyMT; ApcMin/+ cells (from their earlier study) and highlight that Apc has beta-catenin independent functions. Is this phenomenon unique to these two cell lines? Or is it also observed in other TNBC cell lines? A TopFlash reporter assay or detection of active beta-Catenin by a western blot in a few more TNBC cell lines is suggested. We have shown in the two cell lines mentioned, and confirmed in a couple of other TNBC lines (MDA-MB-231 and SUM159) with siRNA-mediated APC knockdown. As mentioned above, we did not include these cell lines in the manuscript focused on PTX resistance. The reason being that we are currently pursuing CRISPR-mediated APC knockout, and because we are focused on the metaplastic subtype of TNBC.

2. Minor corrections:
2.1 The reference (7) in line 302 should be corresponding to your earlier article, “Apc Mutation Enhances PyMT-Induced Mammary Tumorigenesis” (fig S3) and not “APC selectively mediates response to chemotherapeutic agents in breast cancer” We apologize for the oversight. This has been changed.

2.2 A brief description of the TopFlash reporter assay could be included in the Methods section. In addition to the reference, there is now a brief description of the reporter assay included.
