# Supplementary material for: Adenomatous Polyposis Coli loss controls cell cycle regulators and response to paclitaxel in MDA-MB-157 metaplastic breast cancer cells
Source: PLoS One. 2021 Aug 9;16(8):e0255738. doi: 10.1371/journal.pone.0255738 (PMC8351968; doi:10.1371/journal.pone.0255738)
Supplement: Attachment — Submitted filename: Response to Reviewers.docx [file pone.0255738.s008.docx]

**Editorial Review**

Please review your reference list to ensure that it is complete and correct. If you have cited papers that have been retracted, please include the rationale for doing so in the manuscript text, or remove these references and replace them with relevant current references. Any changes to the reference list should be mentioned in the rebuttal letter that accompanies your revised manuscript. If you need to cite a retracted article, indicate the article’s retracted status in the References list and also include a citation and full reference for the retraction notice. The references have been confirmed and no changes were necessary.

**Reviewer #2:**

The authors of the manuscript have mostly addressed the reviewer's comments. I believe the authors would take into consideration the reviewers suggestions for further studies and publications. We appreciate the comments.

**Reviewer #3:**

1. The title of the manuscript is still not justified since the study has been conducted on a single cell line. Either, the authors are encouraged to use multiple cell lines to justify the current title or include MDA-MB-157 in the title itself. The title has been amended to reflect the MDA-MB-157 cells being used in this study.

2. The heading of paragraph "APC shRNA1 cells have increased G2/M arrest after PTX treatment compared to MDA-MB-157" (lines 222 and 223) is not justified because it contradicts with the results from the cell cycle analysis experiments (Fig 1) and your own interpretations (please see lines 30 to 32 and again lines 234 to 236). Therefore, authors are encouraged to either perform more cell cycle experiments to get better standard deviation values to justify their claim or rephrase the heading. Thank you for the observation. The heading has been revised to indicate that APC status (in the MDA-MB-157 cells) doesn’t impact the ability of PTX to induce G2/M arrest.

3. If TopFlash reporter assays were performed in other cell lines (MDA-MB-231 and SUM159) in the current study then those results need to be included in Fig S3 or appropriately cited if they are from earlier work. Thank you for acknowledging the important work done here. Given that this paper is focused on the MDA-MB-157 human metaplastic breast cancer cell line (reflected in the title change also), we do not feel that it’s fitting to include the data on reporter activity in other cell lines. There is no discussion in the paper on this lack of Wnt activity being universal (or in other TNBC cell lines), so we are following the PLOS data policy of sharing all data.
